# Supplementary material for: Warwick-India-Canada (WIC) global mental health group: rationale, design and protocol
Source: BMJ Open. 2021 Jun 11;11(6):e046362. doi: 10.1136/bmjopen-2020-046362 (PMC8202113; doi:10.1136/bmjopen-2020-046362)
Supplement: Supplementary data [file bmjopen-2020-046362supp001.pdf]

Supplementary Material – WIC

Section A – *Work Package 2:- Protocol-based assessment and management of FEP*

Study Population

Patients with 'untreated' FEP and their carers visiting psychiatry department at respective collaborating sites, fulfilling the study inclusion/exclusion criteria will be recruited into the study. Participating patients must be aged 16–45 years with a diagnosis of FEP based on the International Classification of Diseases, 10th revision (ICD-10) codes for schizophrenia (F20), persistent delusional disorder (F22), acute and transient psychotic disorders (F 23), schizoaffective disorder (F25), other and unspecified psychosis (F28-29), mania with psychotic symptoms (F30.2), bipolar affective disorder, current episode manic with psychotic symptoms (F 31.2), severe depressive episode with psychotic symptoms (F32.3) and recurrent depressive disorder and/or current episode depression with psychotic symptoms (F 33.3). Untreated is defined as not having received anti-psychotic medication for more than 30 days since the onset of psychosis. Patients must be able to communicate in Tamil or English in Chennai, and Hindi or English in New Delhi. Study exclusion criteria are: those with overt learning disability (equivalent to IQ <70, ascertained either by previous diagnosis or *detailed history from the patients and their caregivers, corroborated by observations from academic performance assessed using test scores from various subjects (such as language and mathematics)*, organic brain damage and epilepsy of pervasive developmental disorder.

Written informed consent will be obtained from interested and eligible participants prior to participation. Consent will also be sought from interested carers of study participants for carer-reported measures.

Study Assessment Tools

Various clinical scales will be used to assess several domains of symptom severity and patient functioning at baseline and follow-up assessments. Baseline assessment will be conducted soon after entry into the service, and follow-up assessments will be conducted at 3, 6 and 12 months.

Socio-demographic, clinical, and socio/family variables will be collected using semi-structured (MRC demographic schedule) face-to-face clinical interview. Psychopathology will be assessed using the: (i) Scale for the Assessment of Positive Symptoms (SAPS) (1); (ii) Scale for the Assessment of Negative Symptoms (SANS) (1); (iii) Young Mania Rating Scale (YMRS) (2); (iv) Montgomery - Asberg Depression

Singh SP, *et al. BMJ Open* 2021; 11:e046362. doi: 10.1136/bmjopen-2020-046362

Rating Scale (3); and (v) Brief Psychiatric Rating Scale (BPRS) (4). Severity of illness and functioning will be measured using the Global Assessment of Functioning (GAF) Scale (5), the Social And Occupational Functioning Assessment Scale (SOFAS) (6) and 5-level EQ-5D (EQ 5 D 5L) (7). Other assessment tools that will be used in this study are: (i) The Emerging Psychosis Attribution Schedule (EPAS) to assess patient and carer attributions of symptoms in the emerging phase of a psychotic episode (8); (ii) the Nottingham Onset Schedule (NOS) to establish duration of untreated psychosis (9); (iii) an amended version of the Encounter form to assess service encounters during the psychotic phase of the illness (8); (iv) Medication Adherence Rating Scale (MARS) for assessment of medication adherence in psychosis(10); (v) an assessment schedule to assess the nature of insight in psychosis (11); (vi) tools for assessment of risk factors (trauma exposure) including the Childhood Trauma Questionnaire (CTQ) (12), Harvard Trauma Questionnaire (HTQ) (13) and the List Of Threatening Experiences (LTE) (14); and (vii) Brief Assessment of Cognition in Schizophrenia (BACS) for neurocognition. Additionally, we will also capture data on treatment, relapse and physical health. The caregivers will be assessed on the Burden Assessment Schedule (BAS) (15). Engagement of caregivers will be encouraged to obtain accurate patient-information including patients' medical history, risk factors, family responses to illness, help-seeking and potential impact on family. *The estimated time to complete the study assessments will be around 60 min - 90 min. Each assessment will be conducted in two or more sessions depending on the participant's time, availability and comfort.* Supplementary table 1 depicts the assessment schedule for both baseline and follow-up assessments.

Intervention and Management of FEP

Management of FEP

Following a comprehensive assessment as described above, patients with FEP will be offered the following treatment options:

Antipsychotics

- Oral antipsychotics in lowest possible dose taking into account of effectiveness, tolerability and patient preference.
- Depot antipsychotics may be used if adherence is a significant problem or based on patient preference.

- The choice of antipsychotic medication will be discussed by the service user and healthcare professional, and decision will be made, taking into account of the views of the carer. The study team will provide more information on antipsychotics of choice and discuss the likely benefits and possible side effects of each drug, including:
    - metabolic (including weight gain and diabetes)
    - extrapyramidal (including akathisia, dyskinesia and dystonia)
    - cardiovascular (including prolonging the QT interval)
    - hormonal (including increasing plasma prolactin)
    - other (including unpleasant subjective experiences)

Before starting antipsychotic medication, the study team will undertake and record the following baseline investigations:

- weight and BMI
- waist circumference
- pulse and blood pressure
- fasting blood glucose, glycosylated haemoglobin (HbA_{1c}), blood lipid profile and prolactin levels
- assessment of any movement disorders
- assessment of nutritional status, diet and level of physical activity.
- Before starting antipsychotic medication, offer the person with psychosis or schizophrenia an electrocardiogram (ECG) if:
- specified in the summary of product characteristics (SPC)
- a physical examination has identified specific cardiovascular risk (such as diagnosis of high blood pressure)
- there is a personal history of cardiovascular disease

Psychosocial interventions

For Patients

- Psycho-education
- Counselling
- Supportive psycho therapy
- Cognitive behavioral therapy

Singh SP, *et al. BMJ Open* 2021; 11:e046362. doi: 10.1136/bmjopen-2020-046362

- Relapse prevention therapy
- Group therapy
- Vocational/occupational therapy
- Net working

For Family

- Psycho education
- Family intervention
- Family counselling
- Family therapy

Monitoring and follow-up

- Patients will be assessed formally 3, 6 and 12 months according to the schedule in Fig 1.
- Response to treatment, including symptom and behavioural change, adherence to treatment, relapse, crises and hospitalisations will be recorded at every follow-up
- Weight, BMI, blood pressure, blood glucose and lipids will be monitored at 3, 6 and 12 months and preventive strategies implemented (life style and dietary change, metformin etc.)

Supplementary Table 1: Study Assessment Schedule of WIC

Study Instruments	Baseline	Month 3	Month 6	Month 12
MRC Sociodemographic Schedule	X	-	-	-
Nottingham Onset Schedule (NOS)	X	-	-	-
Emerging Psychosis Attribution Schedule (EPAS)	X	-	-	-
Service Encounters Interview	X	-	-	-
Scale for Assessment of Positive Symptoms (SAPS)	X	X	X	X
Scale for Assessment of Negative Symptoms (SANS)	X	X	X	X
Young Mania Rating Scale (YMRS)	X	X	X	X
Montgomery-Asberg Depression Rating Scale	X	X	X	X
Brief Assessment of Cognition in Schizophrenia (BACS)	X	X	X	X
Global Assessment of Functioning(GAF)	X	X	X	X
Social and Occupational Functioning Assessment Scale (SOFAS)	X	X	X	X
Brief Psychiatric Rating Scale (BPRS)	X	X	X	X
Medication Adherence Rating Scale (MARS)	X	X	X	X
EQ-5D 5 L	X	X	X	X
Burden Assessment Schedule (BAS) – Carer	X	X	X	X
Risk Factor Assessment (CTQ, HTQ & LTE)	X	-	-	-
Physical Health Assessment, treatment, relapse*	X	X	X	X
Analysis of Cost*	X	X	X	X

**Investigator designed ; CTQ, childhood trauma questionnaire; HTQ, Harvard trauma questionnaire; LTE, list of threatening experiences (LTE)*

Section B – <u>Work Package 4:- Mobile technology to ensure continuity of care (Commissioned Project)</u>

Background

In India, over 90% of patients stay in the community with families, and in the absence of state provision, families initiate treatment, procure and supervise medicines and provide psychosocial support including opportunities for education, vocation and recreation (16). Importantly, the stress of caregiving adversely affects carer's' physical and mental health(17). In this WP, we will deliver a home-based psycho-social recovery care model, strategies for reducing carer burden, and evaluate its clinical and cost-effectiveness using a three-pronged approach (Supplementary figure 2). We will deliver a mobile application for home-based psychosocial recovery, strategies for reducing carer burden, and determine its clinical and cost-effectiveness.

In brief, first, we will conduct focus group discussions with patients with schizophrenia, their families/caregivers and mental health professionals, to gather information on their healthcare needs and expected outcomes from a home-based psycho-social care model. Second, utilizing insights from these focus groups and an extensive literature review, we will develop a 'bespoke' home-based psycho-social care model (named as SAKSHAM, meaning effort in Hindi, booklet version) aimed at improving functioning and recovery of 'difficult-to-treat' patients with schizophrenia; and at reducing carer burden.

The Saksham will have two parts: booklets and mobile app. These will be developed after conducting focussed group discussions (FGDs) with stakeholders: patients, family caregivers and mental health professionals. Based on existing evidence and themes developed from the FGDs, the domains identified for the psychosocial intervention were medication adherence, physical health monitoring, activities of daily living, instrumental activities of daily living, diet management and need for psychoeducation. Intervention package for all six target domains will be developed. The content of the Saksham booklets will be in Hindi and English for both patients and caregivers. The content validity of the booklets will be tested by getting inputs from the stakeholders. The content of the booklets will be digitised for the Saksham mobile application. Finally, we will evaluate the feasibility and effectiveness of both the booklet version and mobile based application in a small group of patients with schizophrenia and their caregivers, and will compare against treatment as usual

group. This study will be conducted at AIIMS, New Delhi, India. For an overview of study inclusion/exclusion criteria of study cohort and list of study instruments with assessment schedule, see Supplementary Table 2 and Supplementary Table 3, respectively.

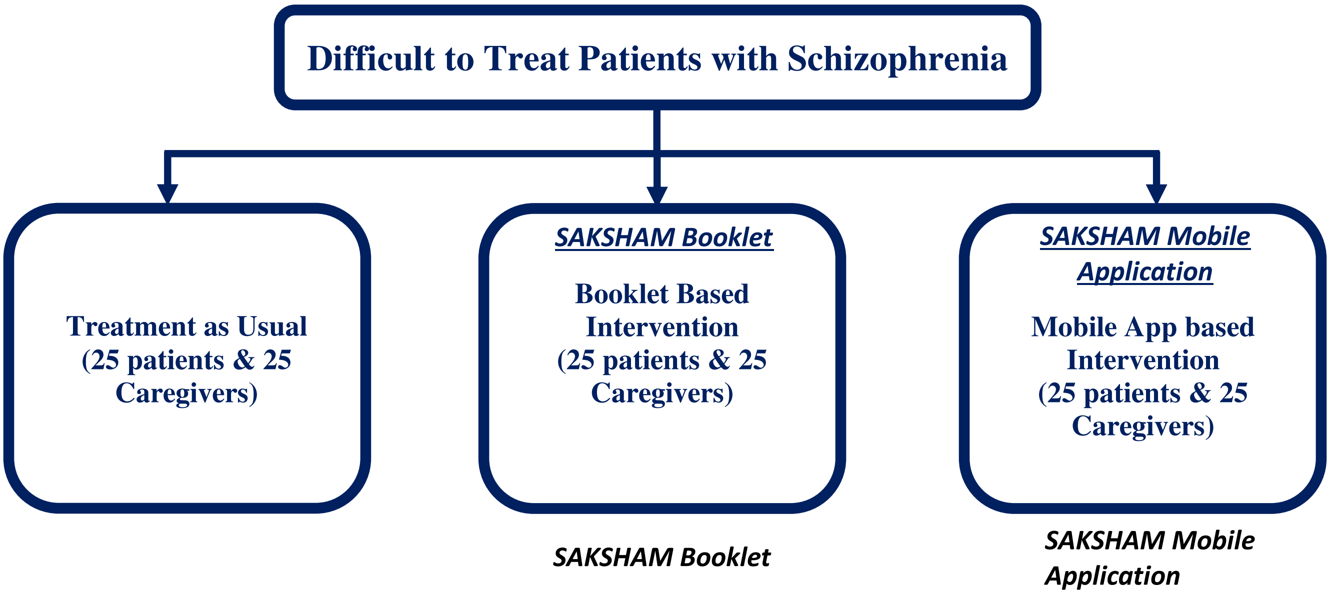

Supplementary Figure 1: Three arm study to evaluate the feasibility and effectiveness of home-based psycho-social recovery model.

BMJ Publishing Group Limited (BMJ) disclaims all liability and responsibility arising from any reliance placed on this supplemental material which has been supplied by the author(s)

Supplementary Table 2 : Study Inclusion /Exclusion Criteria for Patients and their Caregivers.

Patients	Caregivers
Inclusion Criteria	***Inclusion Criteria***
Aged 18–65 years	Immediate family member fulfilling any three of following criteria:<br>• Spending maximum time with the patient.<br>• Supporting the patient financially<br>• Most involved in the care of the patient<br>• Able to be contacted by treatment staff in case of an emergency
Diagnosis of schizophrenia based on International Classification of Diseases - Tenth Revision (ICD-10)codes [Schizophrenia (F20), Persistent delusional disorder (F22), Schizoaffective disorder (F25), Other and Unspecified Psychosis (F28 & F29)]	Using mobile device for at least one year
Duration of illness ≥ 2 years	Able to read Hindi or English
Persistent significant psychopathology defined as score of ≥4 (moderate) on items P1-P7 and items N1-N7 items 7, 20, 25 or 34 on Positive and Negative Syndrome Scale (PANSS).	***Exclusion Criteria***
Persistent significant psychopathology and dysfunction, despite receiving regular treatment	Caregivers suffering from psychiatric disorder which might interfere with the care of patients and his/her co-operation during
Using mobile device for at least one year	Not willing to provide informed consent
Able to read Hindi or English	
Exclusion Criteria	
Unable to provide informed consent	

Supplementary Table 3 : Study Assessment Schedule for WP 4

Study Instruments	Baseline	Month 1	Month 3
**Assessment of Patients**			
Socio-demographic & Clinical Characteristics	X	X	X
Assessment of Psychopathology:	X	X	X
Positive and Negative Syndrome Scale	X	X	X
Schizophrenia Cognitive Rating Scale (SCoRS)	X	X	X
Assessment of Physical Health	X	X	X
Assessment of Treatment Adherence:	X	X	X
Medication Adherence Rating Scale (MARS)	X	X	X
Assessment of Functioning:	X	X	X
Katz Index of Independence in Activities of Daily Living (ADL)	X	X	X
Lawton Brody Instrumental Activities of Daily Living (IADL)	X	X	X
World Health Organization Disability Assessment Schedule (WHODAS)2.0	X	X	X
Clinical Global Improvement	X	X	X
Assessment of Caregivers			
Mini International Neuropsychiatric Interview (MINI) Screen for caregivers	X	X	X
Family Burden Interview Schedule	X	X	X
Ways of Coping Checklist-Hindi Adaptation (WCC-HA)	X	X	X

Singh SP, *et al. BMJ Open* 2021; 11:e046362. doi: 10.1136/bmjopen-2020-046362

Section C – Work Package 5:-Early detection using a community awareness programme (Commissioned Project)

India has a young population – over half of Indian's population are under the age of 30 years (22), with an overall mental illness prevalence rate of 10.6% and nearly 150 million Indians in need of active interventions (23). Taking into account that 80% of FEP usually occurs at a very young age (i.e. between 16 and 30 years of age) (24) and that LMICs have predominantly young populations, it is highly likely that an overwhelming majority of people experiencing FEP live in LMIC countries such as India. However, lack of adequate mental health services in India along with other impediments to care such as stigma and lack of awareness can lead to delayed help-seeking contributing to long DUPs. A longer duration of DUP is associated with a poor treatment response, greater subsequent disability and higher mortality rates (25); therefore, timely identification and diagnosis are pivotal. India does not have standardized early detection and intervention programs for psychotic disorders. There are sporadic awareness and screening programs held in some schools and colleges. Moreover, these programs have not integrated active referral of "cases" to appropriate interventions. This work package therefore aims to increase mental health literacy of teachers and students; to use a web-based screening tool to detect youth who could benefit from mental health services; and to refer such youth to appropriate services.

Intervention

We will develop mental health training modules for teachers and mental health awareness modules for students. The training manual for teachers will contain information on mental illness, especially psychosis and is aimed to create awareness about the early signs and symptoms of psychosis to facilitate early referrals to clinical care. Data on Knowledge, Attitude and Practice (KAP) of the teachers and students will be obtained at baseline, month 12 and month 24. Training for teachers include one half day session (4 hours) and three subsequent booster sessions (1 hour) in three-month intervals. Students will be provided education on mental health – one half day session (4 hours). Voluntary mental health screening tool with the PHQ-9, GAD-7 and CAPE-P15 will be made available to all students through web-based applications and mobile applications. The overall research methodology is depicted in supplementary figure 3 and for the list of study instruments, see supplementary Table 4.

Singh SP, *et al. BMJ Open* 2021; 11:e046362. doi: 10.1136/bmjopen-2020-046362

Supplementary figure 3: Research Methodology of Work Package 5

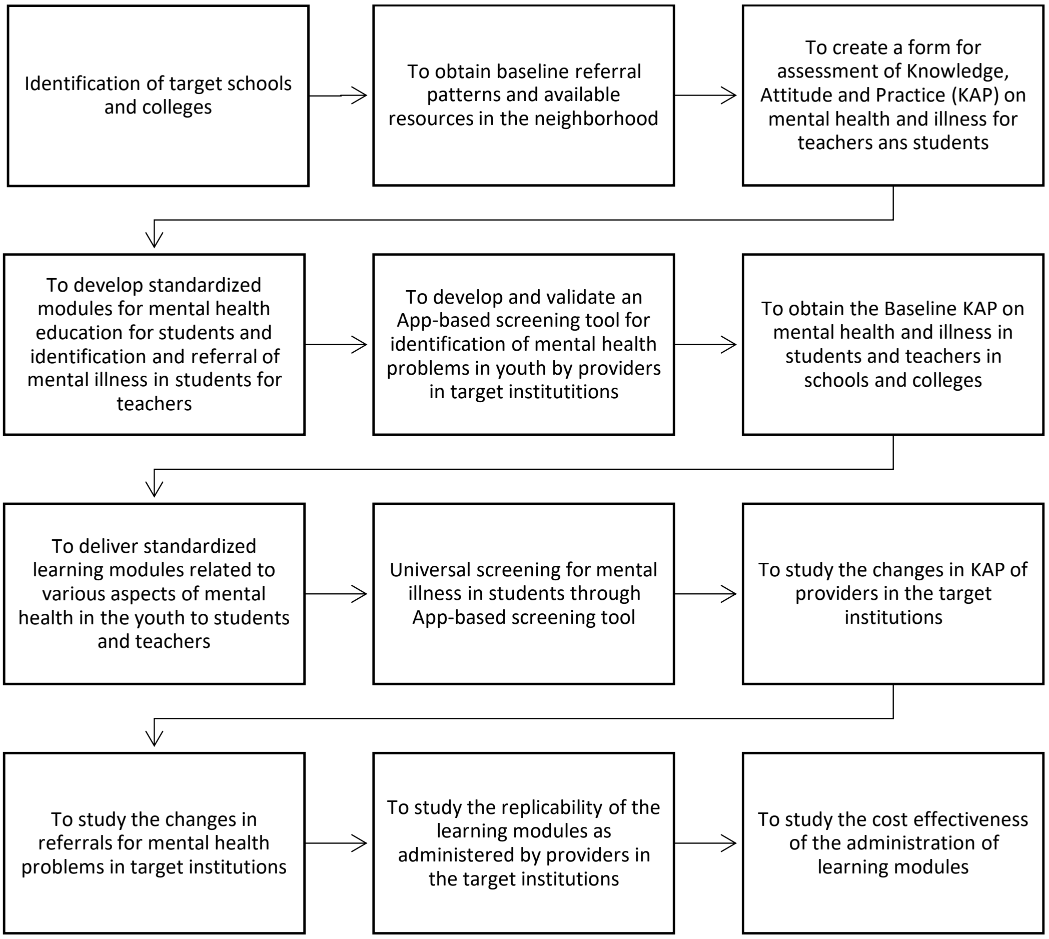

Supplementary Table 4 : List of study instruments

**Knowledge, Attitude and Practice (KAP) questionnaires**
The Mental Health Knowledge Schedule<br>The Reported and Intended Behaviour scale
**Mental health screening tool**
Patient Health Questionnaire-9 (PHQ-9)<br>Generalized Anxiety Disorder-7 scale (GAD-7)<br>Community Assessment of Psychic Experiences – Positive 15-items Scale (CAPE-P15)

Singh SP, *et al. BMJ Open* 2021; 11:e046362. doi: 10.1136/bmjopen-2020-046362

References

1. Andreasen NC, Arndt S, Miller D, Flaum M, Nopoulos P. Correlational studies of the Scale for the Assessment of Negative Symptoms and the Scale for the Assessment of Positive Symptoms: an overview and update. Psychopathology. 1995;28(1):7-17.
2. Young RC, Biggs JT, Ziegler VE, Meyer DA. A rating scale for mania: reliability, validity and sensitivity. Br J Psychiatry. 1978;133:429-35.
3. Montgomery SA, Asberg M. A new depression scale designed to be sensitive to change. Br J Psychiatry. 1979;134:382-9.
4. Overall JE, Gorham DR. The Brief Psychiatric Rating Scale. Psychological Reports. 1962;10(3):799-812.
5. Jones SH, Thornicroft G, Coffey M, Dunn G. A brief mental health outcome scale-reliability and validity of the Global Assessment of Functioning (GAF). Br J Psychiatry. 1995;166(5):654-9.
6. Skodol AE, Link BG, Shrout PE, Horwath E. Toward construct validity for DSM-III Axis V. Psychiatry Res. 1988;24(1):13-23.
7. Herdman M, Gudex C, Lloyd A, Janssen M, Kind P, Parkin D, et al. Development and preliminary testing of the new five-level version of EQ-5D (EQ-5D-5L). Qual Life Res. 2011;20(10):1727-36.
8. Singh SP, Brown L, Winsper C, Gajwani R, Islam Z, Jasani R, et al. Ethnicity and pathways to care during first episode psychosis: the role of cultural illness attributions. BMC Psychiatry. 2015;15:287.
9. Singh SP, Cooper JE, Fisher HL, Tarrant CJ, Lloyd T, Banjo J, et al. Determining the chronology and components of psychosis onset: The Nottingham Onset Schedule (NOS). Schizophr Res. 2005;80(1):117-30.
10. Vanelli I, Chendo I, Gois C, Santos J, Levy P. [Medication adherence rating scale]. Acta Med Port. 2011;24(1):17-20.
11. David A, Buchanan A, Reed A, Almeida O. The assessment of insight in psychosis. Br J Psychiatry. 1992;161:599-602.
12. Pennebaker JW, Susman JR. Disclosure of traumas and psychosomatic processes. Soc Sci Med. 1988;26(3):327-32.
13. Mollica RF, Caspi-Yavin Y, Bollini P, Truong T, Tor S, Lavelle J. The Harvard Trauma Questionnaire. Validating a cross-cultural instrument for measuring torture, trauma, and posttraumatic stress disorder in Indochinese refugees. J Nerv Ment Dis. 1992;180(2):111-6.
14. Brugha TS, Cragg D. The List of Threatening Experiences: the reliability and validity of a brief life events questionnaire. Acta Psychiatr Scand. 1990;82(1):77-81.
15. Thara R, Padmavati R, Kumar S, Srinivasan L. Instrument to assess burden on caregivers of chronic mentally ill. Indian J Psychiatry. 1998;40(1):21-9.
16. Chadda RK. Caring for the family caregivers of persons with mental illness. Indian J Psychiatry. 2014;56(3):221-7.
17. Chadda RK, Singh TB, Ganguly KK. Caregiver burden and coping: a prospective study of relationship between burden and coping in caregivers of patients with schizophrenia and bipolar affective disorder. Soc Psychiatry Psychiatr Epidemiol. 2007;42(11):923-30.
18. Stalin P, Abraham SB, Kanimozhy K, Prasad RV, Singh Z, Purty AJ. Mobile Phone Usage and its Health Effects Among Adults in a Semi-Urban Area of Southern India. J Clin Diagn Res. 2016;10(1):LC14-6.
19. Firth J, Torous J. Smartphone Apps for Schizophrenia: A Systematic Review. JMIR Mhealth Uhealth. 2015;3(4):e102.

20. Sood M, Chadda RK, Singh P. Mobile health (mHealth) in mental health: Scope and applications in low-resource settings. Natl Med J India. 2016;29(6):341-3.
21. M Sood RC, K Sinha Deb, R Bhad , A Mahapatra, R Verma, AK Mishra. Scope of Mobile Phones in Mental Health Care in Low Resource Settings. Journal of Mobile Technology in Medicine. 2016(5:2:33–37).
22. Factbook CIAW. India Demographics Profile 2018 2018 [updated January 20 2018. Available from: https://www.indexmundi.com/india/demographics_profile.html.
23. Murthy RS. National Mental Health Survey of India 2015-2016. Indian J Psychiatry. 2017;59(1):21-6.
24. Shiers D, Lester H. Early intervention for first episode psychosis. BMJ. 2004;328(7454):1451-2.
25. Farooq S, Large M, Nielssen O, Waheed W. The relationship between the duration of untreated psychosis and outcome in low-and-middle income countries: a systematic review and meta analysis. Schizophr Res. 2009;109(1-3):15-23.
