# Supplementary material for: Warwick-India-Canada (WIC) global mental health group: rationale, design and protocol
Source: BMJ Open. 2021 Jun 11;11(6):e046362. doi: 10.1136/bmjopen-2020-046362 (PMC8202113; doi:10.1136/bmjopen-2020-046362)
Supplement: Author's manuscript [file bmjopen-2020-046362.draft_revisions.pdf]

BMJ Open is committed to open peer review. As part of this commitment we make the peer review history of every article we publish publicly available.

When an article is published we post the peer reviewers' comments and the authors' responses online. We also post the versions of the paper that were used during peer review. These are the versions that the peer review comments apply to.

The versions of the paper that follow are the versions that were submitted during the peer review process. They are not the versions of record or the final published versions. They should not be cited or distributed as the published version of this manuscript.

BMJ Open is an open access journal and the full, final, typeset and author-corrected version of record of the manuscript is available on our site with no access controls, subscription charges or pay-per-view fees (http://bmjopen.bmj.com).

If you have any questions on BMJ Open's open peer review process please email info.bmjopen@bmj.com

BMJ Open

The Warwick-India-Canada (WIC) Global Mental Health Group: Rationale, Design and Protocol.

Journal:	*BMJ Open*
Manuscript ID	bmjopen-2020-046362
Article Type:	Protocol
Date Submitted by the Author:	29-Oct-2020

Complete List of Authors:	Singh, Swaran; University of Warwick Warwick Medical School<br>Mohan, Mohapradeep; University of Warwick Warwick Medical School,<br>Iyer, Srividya ; McGill University,<br>Meyer, Caroline; University of Warwick,<br>Currie, Graeme; University of Warwick<br>Shah, Jai; McGill University, Department of Psychiatry, McGill University<br>Madan, Jason; University of Warwick Warwick Medical School, Warwick Medical School<br>Birchwood, Max; University of Warwick Warwick Medical School, Division of mental health & well-being<br>Sood, Mamta; All India Institute of Medical Sciences, Department of Psychiatry<br>Padmavarti, Ramachandran; Schizophrenia Research Foundation<br>Chadda, Rakesh; All India Institute of Medical Sciences, Psychiatry<br>Lilford, RJ; University of Birmingham,<br>Thara, Rangaswamy; Schizophrenia Research Foundation<br>Furtado, Vivek; University of Warwick Warwick Medical School, Mental Health and Wellbeing; Birmingham and Solihull Mental Health NHS Foundation Trust, Forensic Psychiatry

Keywords:	Schizophrenia & psychotic disorders < PSYCHIATRY, Child & adolescent psychiatry < PSYCHIATRY, Adult psychiatry < PSYCHIATRY

SCHOLARONE™
Manuscripts

I, the Submitting Author has the right to grant and does grant on behalf of all authors of the Work (as defined in the below author licence), an exclusive licence and/or a non-exclusive licence for contributions from authors who are: i) UK Crown employees; ii) where BMJ has agreed a CC-BY licence shall apply, and/or iii) in accordance with the terms applicable for US Federal Government officers or employees acting as part of their official duties; on a worldwide, perpetual, irrevocable, royalty-free basis to BMJ Publishing Group Ltd ("BMJ") its licensees and where the relevant Journal is co-owned by BMJ to the co-owners of the Journal, to publish the Work in this journal and any other BMJ products and to exploit all rights, as set out in our licence.

The Submitting Author accepts and understands that any supply made under these terms is made by BMJ to the Submitting Author unless you are acting as an employee on behalf of your employer or a postgraduate student of an affiliated institution which is paying any applicable article publishing charge ("APC") for Open Access articles. Where the Submitting Author wishes to make the Work available on an Open Access basis (and intends to pay the relevant APC), the terms of reuse of such Open Access shall be governed by a Creative Commons licence – details of these licences and which Creative Commons licence will apply to this Work are set out in our licence referred to above.

Other than as permitted in any relevant BMJ Author's Self Archiving Policies, I confirm this Work has not been accepted for publication elsewhere, is not being considered for publication elsewhere and does not duplicate material already published. I confirm all authors consent to publication of this Work and authorise the granting of this licence.

For peer review only

The Warwick-India-Canada (WIC) Global Mental Health Group: Rationale, Design and Protocol.

Swaran Preet Singh*,[1] Mohapradeep Mohan*,[1] Srividya N Iyer, [2] Caroline Meyer,[3] Graeme Currie,[4] Jai Shah,[2] Jason Madan,[5] Maxmillan Birchwood,[1] Mamta Sood,[6]; Padmavati Ramachandran,[7] Rakesh K Chadda, [6] Richard J Lilford,[8] Thara Rangaswamy, [7] Vivek Furtado, [1] and on behalf of the WIC Consoritum

** S.P.S and MM contributed equally*

Author Affiliations

(1) Division of Mental Health and Wellbeing, Warwick Medical School, University of Warwick, Coventry, UK

(2) Douglas Mental Health University Institute, McGill University, Montreal, Canada

(3) WMG and Warwick Medical School, University of Warwick, Coventry, UK

(4) Warwick Business School, University of Warwick, Coventry, UK

(5) Warwick Clinical Trials Unit, Warwick Medical School, University of Warwick, Coventry, UK

(6) Department of Psychiatry, All India Institute of Medical Sciences, rg, Ansari Nagar,, New Delhi, India.

(7) Schizophrenia Research Foundation, R/7A, North Main Road, Anna Nagar West Extension, Chennai, Tamil Nadu, India

(8) Institute of Applied Health Research, University of Birmingham, Birmingham, UK.

Word Count : 4168

Corresponding Author

Prof Swaran Preet Singh, Division of Mental Health and Wellbeing, Warwick Medical School, University of Warwick, Coventry, UK.

Email: S.P.Singh@warwick.ac.uk

For peer review only - http://bmjopen.bmj.com/site/about/guidelines.xhtml

Abstract

Introduction

The primary aim of the NIHR funded global health research group, Warwick-India-Canda (WIC), is to reduce the burden of psychotic disorders in India. India has a large pool of undetected and untreated patients with psychosis and a treatment gap exceeding 75%. Evidence-based packages of care have been piloted, but delivery of treatments still remains a challenge. Even when patients access treatment, there is no continuity of care. The overarching ambition of WIC programme is to improve patient outcomes through: (i) developing culturally tailored clinical interventions ; (ii) early identification and timely treatment of individuals with mental illness; and (iii) improving access to care by exploiting the potential of digitial technologies.

Methods and Analysis

This multicentre, multi-component research programme, comprising five work packages (WPs) and two cross-cutting themes(CTs), is being conducted at two settings: Schizophrenia Research Foundation (SCARF), Chennai (South India) and All India Institute of Medical Sciences (AIIMS), New Delhi (North India). WIC will: (i) develop and evaluate evidence-informed interventions for early and first-episode psychosis; (ii) determine pathways of care for early psychosis; (iii) investigate the efficacy and cost-effectiveness of community care models, including digital and mobile technologies; (iv) develop strategies to reduce the burden of mental illnesses among youth; (v) assess the economic burden of psychosis on patients and their carers; and (vi) determine the feasibility of an early intervention in psychosis programme in India.

Ethics and Dissemination

This study has been approved by the University of Warwick's Biomedical and Scientific Research Ethics Committee (reference: REGO-2018-2208), Coventry, UK and research ethics committees of all participating organisations. Research findings will be disseminated through peer-reviewed scientific publications, presentations at learned societies, and visual media.

Keywords: First episode psychosis, Schizophrenia, Mental health, Low-and-middle-income countries, NIHR Global Health Research Group, WIC

For peer review only - http://bmjopen.bmj.com/site/about/guidelines.xhtml

Article Summary

Strengths and Limitations of this Study

- We will identify barriers and facilitators for implementing early intervention services in psychosis in India, and contextually tailor programmes with proven efficacy for applicability in the developing world such as India, both for improving clinical care, and expanding availability to communities with limited access to care.
- WIC will build research capacity relevant to global mental health, including in methodologies such as health economic evaluation and implementation science.
- We will develop innovative community-based models for continuity of clinical care for difficult-to-treat cases of schizophrenia and early detection of emerging disorders.
- Some studies embedded in this research programme are exploratory and hypothesis generating, therefore large studies are warranted for conclusive evidence.

For peer review only

For peer review only - http://bmjopen.bmj.com/site/about/guidelines.xhtml

Introduction

Globally, psychotic disorders such as schizophrenia, are among the top 20 leading contributors to disability [1]. Nearly 80% of individuals develop their first episode of psychosis (FEP) during adolescence or early adulthood [2]. Importantly, the burden of disease and disability is particularly high in low-and-middle-income countries (LMICs) [3]. LMICs' have predominantly young populations, with numerous FEP cases without adequate care [3-5]. Despite efforts to develop healthcare models for accessible and effective care in LMICs, the implementation of such models remains a major challenge [6].

In the developed world, among the most significant reforms since deinstitutionalisation, has been the establishment of early intervention in psychosis services (EIS) [7]. Given their clinical and cost-effectiveness [8-10], many countries have scaled up EIS, as part of their mental health policy. EIS focus on reducing the duration of untreated psychosis (DUP), provision of holistic and evidence-based specialist care during the early 'critical period' of psychosis and interventions for individuals who are at high risk of developing a psychotic disorder (clinical high risk or ultra-high risk) [11 12]. Despite such promising evidence, many LMICs have not implemented EIS into their mental health systems. Inadequate mental health workforce, fragmented healthcare systems [13], and scarcity of research and implementation capacity are significant barriers to introducing such care in LMICs [14 15]. Nonetheless, DUP is significantly higher in LMICs [16], and therefore EIS provision is potentially of great value. Help-seeking in LMICs is often pluralistic, with traditional practitioners being the initial, and sometimes the only source of care[17]. Therefore, a simple 'transplantation' of the 'Western' EIS model to LMIC context is neither feasible nor practical. However, it may be possible to incorporate the principles and therapeutic components of EIS into locally contextualised mental health care in LMICs, [3 18]. This is the key premise underpinning the WIC project.

Aim and Strategic Goals

The National Institute of Health Research (NIHR) Global Health Research Group (GHRG) on Psychosis Outcomes: the Warwick-India-Canada Network (WIC) aims to reduce the burden of psychotic disorders and improve health, wellbeing and functioning of those with psychotic disorders in India. Informed by principles of evidence-based EIS developed in the UK and Canada, the WIC GHRG will develop culturally sensitive interventions that will expand mental health services to

For peer review only - http://bmjopen.bmj.com/site/about/guidelines.xhtml

communities that currently have limited access in India. WIC will also build and sustain much-needed research capacity and ensure the implementation of evidence-based practice systematically.

The Indian Context

India with its 1.3 billion population, has a severe shortage of mental health care provision. Compared with a global average of 3.96 psychiatrists/ 100,000 population, India has 0.75 psychiatrists/100,000 population. The overall availability of mental healthcare workers (psychiatrists, clinical psycologists, and psychiatric social workers) is <1/ 100,000 population. [19 20]. India therefore has a large number of patients with undetected, untreated or inadequately treated patients with psychotic disorders [21]. Even when patients access care, there is no continuity; treatment is sporadic and focused on symptomatic control and crisis management rather than recovery [22]. Social stigma and discrimination associated with mental illness are major impediments to delivering evidence-based care [23]. Studies have found that many prodromal patients ('at-risk') remain undetected in the community[24]; and there are no early identification programmes in the community.

Objectives

The key objectives of WIC are to:

a) develop and evaluate evidence-informed, culturally tailor-made interventions to make sustained health improvements for poorly served individuals with a first episode of psychosis;
b) understand pathways of care to develop early detection strategies for psychosis;
c) create a dataset of well-characterised inception cohorts of FEP with research-ready systems and processes, that will enable answer new research questions;
d) investigate clinical and cost-effectiveness of innovative community care models for psychosis including digital and mobile applications;
e) develop and implement a school and college based intervention programs for early detection and referral of individuals with psychosis; and
f) build research capacity by providing a strong multidisciplinary research and training programme for researchers based in India.

A larger goal is to build strong research collaborations between India, UK and Canada partners for long-term research, training, knowledge exchange and service imporvement.

For peer review only - http://bmjopen.bmj.com/site/about/guidelines.xhtml

Methods

WIC Programme - Design

The design of WIC programme is depicted in Figure 1. Our strategic plan comprises five work packages (WP) and two cross-cutting themes (CTs) with clear objectives and deliverables. Additionally, we will exploit the potential of digital/mobile technologies to: (i) identify mental disorders in students, and (ii) increase access to clinical services and continuity of care for difficult-to-treat cases of schizophrenia. The study will be conducted in accordance with ethical principles that comply with the Declaration of Helsinki and the ICH Harmonised Tripartite Guideline for Good Clinical Practice. All participants will provide written informed consent prior to entering a study.

WIC Programme - Management

The University of Warwick is the sponsor organisation. A programme manager (PM) is responsible for the day-to-day management of the research programme and supports the GHRG director for the overall delivery of the research programme. The PM, GHRG director and work package leads are responsible for the overall management of the study and meet monthly by video conference chaired by the director. The programme steering committee (PSC), comprising PM, GHRG director, co-investigators and WP leads provide overall strategic direction, ensure good governance, provide scientific input, guide capacity-building activities and provide financial probity when required. An independent external scientific advisory group (ESAG) comprising five international experts will monitor and assess the study conduct and progress. Both PSC and ESAG meet annually with the consortium to assess the scientific quality of research and to give feedback.

Study Sites

WIC brings together four institutions: University of Warwick, UK; McGill University, Canada; All India Institute of Medical Sciences (AIIMS), New Delhi, India; and Schizophrenia Research Foundation (SCARF), Chennai, India. SCARF, based in Chennai, is a non-profit voluntary organisation providing care to patients with psychosis and a World Health Organisation (WHO)-collaborating centre for schizophrenia research. With a population of over 7 million, Chennai is the fourth largest metropolis in India. AIIMS, based in New Delhi with its metropolitan area population of 30 million, is a tertiary care hospital involved in providing undergraduate and postgraduate medical education and research, and hosts the National Drug Dependance Treatment Centre, a WHO collaborating centre.

For peer review only - http://bmjopen.bmj.com/site/about/guidelines.xhtml

Work Package 1:- Partnerships, collaborations and stakeholder engagement

WP-1 will develop a network of external stakeholders in youth mental health including user and carer advocacy groups, clinicians, academicians and policy makers, and will be conducted at both study sites. A situational and needs analyses will be conducted to identify user and carer priorities, barriers to help-seeking and modifiable aspects of care pathways. 'Therapeutic ingredients' of early intervention that can be introduced in clinical care in India will be identified.

Work Package 2:- Protocol-based assessment and management of FEP

Utilising the insights from WP1 and evidence-based interventions developed in the UK and Canada for EIS, in WP2 we will adapt protocols from the UK NEDEN study [25], the largest FEP cohort in the world, to gather FEP cases at both SCARF and AIIMS, ensuring valid and reliable translation of assessment tools. A comprehensive package of biopsychosocial care, tailored to the local context will be developed and delivered to all new FEP cases and their short and medium-term outcomes measured. An FEP patient registry (affective and nonaffecrtive psychosis) will be maintained at our study sites. The study population, study assessment tools and study assessment schedules are described in Section A in the supplementary material.

Work Package 3:- Understanding pathways to care

The objective of WP3 is to determine pathways to care in FEP at both sites, using a combination of qualitative and quantitative approaches. We will explore DUP and its components (help-seeking delay, treatment delay following presentation) , cultural attribution of psychosis symptoms (e.g., individual, supernatural), help-seeking behaviour and modifiable factors that contribute to DUP. We will exploit the established methodology for case-ascertainment and care pathways from the NIHR ENRICH Programme [26]. We will examine the following questions:

I. Do the two cohorts (South India Vs North India) significantly differ in culturally mediated illness attributions (i.e., individual, natural, social, supernatural, and no attribution) during FEP? The cohorts vary demographically and in terms of study setting (outpatient NGO in South India vs. large general hospital with emergency, inpatient and outpatient facilities in North India).

II. Do the two cohorts differ in their pathways to care (i.e., DUP, compulsory detention, psychiatrists, emergency services, general practitioner, faith-based organisations) during FEP?

For peer review only - http://bmjopen.bmj.com/site/about/guidelines.xhtml

III. Are the pathways to care during FEP influenced by culturally mediated illness attributions and other relevant socio-demographic (i.e., gender, religious practice, marital status, living alone, education, income) and clinical confounders such as DUP?

Study setting, population and study assessment tools: This study will be conducted at SCARF, Chennai and AIIMS, New Delhi, using the datasets collected from FEP patients recruited as part of WP2. The study instruments that will be used in this study are: (i) *The Emerging Psychosis Attribution Schedule* (EPAS) [27]; (ii) Nottingham Onset Schedule (NOS) [28]; and (iii) an amended version of the *Encounter form* by Gater, Sousa [27].

Work Package 4:- Mobile technology to ensure continuity of care

The objective of WP4 is to develop a home-based psycho-social care model for 'difficult-to-treat' patients with schizophrenia aimed at functioning and recovery, and an associated mobile-based application. We will compare the effectiveness of a paper-version of the model with the mobile app in improving patient outcomes and reducing carer burden. This study will be conducted at AIIMS, New Delhi. The study population, inclusion/exclusion criteria, methodology, assessment tools and study assessment schedules are described in Section B of the supplementary material.

Work Package 5:-Early detection using a community awareness programme

The primary aim of WP5 is to develop and implement a school and college-based intervention for early detection and referral of individuals with emerging mental health problems. We wish to pilot a school- and college-based programme of online screening for emerging mental health problems; assess the effectiveness of using community resources (teachers, peers and youth groups) to provide psycho-social intervention; and determine the clinical and cost effectiveness of the screening programme. This study will be conducted by SCARF, Chennai, India. The identified catchment area for the study is Anna Nagar, Chennai and adjacent areas that have fourteen schools and four colleges. Informed consent from the management of the educational institutions will be sought before conducting the study at their institution. The study assessment tools and study intervention are described in Section C of the supplementary material.

For peer review only - http://bmjopen.bmj.com/site/about/guidelines.xhtml

Two Cross Cutting Themes (CT)

Implementation Science

Despite being the first LMIC to introduce a National Mental Health Programme, there remains a significant implementation gap in the delivery of evidence-based interventions for mental health care in India [29]. [30]. Implementation science represents an approach focused upon understanding the challenges, identifying the barriers and utilizing the facilitators to help translate research-based evidence to 'real-world' clinic and community settings, developing leadership and changing professional practice to support delivery of evidence-based innovation [31].

The primary objective of the WIC implementation science programme is to evaluate the implementation of the early intervention in psychosis programme (EIS) in India. We will provide insight into facilitators and barriers to the development, sustaining and scaling up of the FEP service in India. Our specific aims are (i) to identify barriers and facilitators to implementation, sustaining (beyond pilot) and scaling up of evidence-based early intervention to address psychosis; (ii) to assess how the original intervention is adapted to fit context, to what extent it then diverges from its original evidence base. The study will be carried out at SCARF, Chennai.

Health Economics

The primary objective of the health economics theme is to assess the cost of treatment for patients with FEP attending AIIMS from the payer and provider perspective. We will develop and pilot a questionnaire to determine direct and indirect costs experienced by patients with mental illness, and their households, and the coping strategies used in response to these costs. From these data, we will estimate mean, median and quartile costs at each time point. We will also conduct a bottom-up costing of the typical package of care offered to those participating in WP2 at AIIMS. This will be based on unit cost data collected during the study, along with interviews with clinicians taking part in the study on the care delivered, and the time taken to deliver it.

Capacity Building Activities

There is an urgent need to develop and deliver high quality research capacity-building activities in India, ranging from modules on research methodology to doctoral level training and courses aimed at healthcare and leadership.

For peer review only - http://bmjopen.bmj.com/site/about/guidelines.xhtml

PhDs

WIC, along with TATA Trust, India and Warwick Medical School are supporting three PhD candidates from India at the University of Warwick, whilst enabling them to carry out their primary research activities in their home countries. These three students, who were selected after a competitive process, commenced their doctoral studies in May 2019. The PhD topics included projects that are nested within WIC and stand-alone projects that are closely aligned to the WIC research priorities. At least two University of Warwick-based supervisors and one partner country-based supervisors are allocated to each student for their PhDs. Topics approved for the first cohort of WIC PhD students include: (i) health economics; (ii) pathways of care and service engagements in early intervention services; and (iii) assessing factors associated with hospital re-admissions and strategies to reduce the burden of re-admissions in psychiatric hospitals in India.

Research leadership training programme

The WIC will provide a bespoke strategic leadership programme to clinicians involved in youth mental health in India. At Warwick Business School (WBS), we already have an international distance learning *Leadership Development Programme (LDP)* for clinicians, with case studies from Africa and South East Asia. We will develop a contextualized, distance-learning intervention about leadership and innovation for mental health professionals in India. This innovative training will be focussed on developing another case from the WIC programme, specific to youth mental health in India. Integrating leadership and innovation training in clinical and service development domains will be beneficial for mental health professionals, particularly in formulating clinical priorities and engaging meaningfully with policy makers of the country.

Planned Extensions

The WIC GHRG has secured funding from NIHR to conduct an additional programme of work, which builds on the existing programme of work, for a duration of up to 12 months. In this costed extension programme, we will: (i) extend our current school-based screening for mental disorders (WP5) in two vulnerable populations: a) refugee youth (Jammu & Kashmir); and b) a high-risk tribal population in Mahrashtra; (ii) expand our FEP registry (WP2) to a rural setting (Nagpur), for potential comparisons with other sites and with existing datasets and including additional research questions of interest (such as cardiometabolic parameters, life style interventions); (iii) extend

For peer review only - http://bmjopen.bmj.com/site/about/guidelines.xhtml

health economic analysis to include rural populations and explore how financial burden and coping mechanisms differ between settings; and (iv) develop a research-based leadership training programme.

Analysis plan

For WP-1, a proposective cross-sectional study in the form of interview with mental health professionals will be conducted to capture information on current practices and available resources for the management of FEP. The semi-structred performa will be developed that will capture information on facilities available for FEP management at each health centres, pharmacological and non-pharmacological management, hostpitalization data and follow-up data of FEP cases. The data will be analysed using standard summary statistsical methods.

For the remaining WP's and CTs, we will use standard descriptive statistical methods to evaluate the data. Where appropriate, continuous variables will be assessed for normality, and non-normally distributed variables will be transformed or assessed using non-parametric tests as appropriate. To compare the data (e.g. sociodemographic and clinical factors) between and within study sites, we will use independent sample *t*-tests for continuous variables and χ^2 for dichotomous variables (WP-2 & WP-3). To assess the change in several domains of symptom severity and functioning at pre-specified time points, we will use a general linear modelling analysis, adjusting for various potential confounders (e.g. WP-2, WP-4). We will use test adjusted and unadjusted associations using appropriate statistical tests as appropriate (e.g. WP-3). For unadjusted analysis: (i) ANOVA for testing unadjusted assocaitions between ethinic groups and culturally mediated illnbess attributions and service encounters (normally distributed continuous outcomes); (ii) Kruskal – Wallis rank sum tests (for non-normally distributed continuous outcomes); and (iii) logistic regression for dichotomous variables. For adjusted association analysis, we will use multiple logistic regression analysis, controlling for various confounders of interest. To provide information regarding the magnitude of the change in scores in domains of interest (e.g. WP-5), *Cohen's d* effect sizes will be calculated with effect size values of ≥0.5, ≥0.8 and ≥1.0 corresponding to a moderate, large, and very large effect, respectively n(e.g WP-5). For CTs such as implementation science and health economics, we will use standard descriptive statistics to describe the data and framework analysis (implementation science), if deemed necessary.

For peer review only - http://bmjopen.bmj.com/site/about/guidelines.xhtml

Statistical significance will be inferred at P <0.05. We will consider sensitivity to change as adequate, if the improvement is statistically significant (P<0.05) and with at least a moderate effect size (Cohen's d ≥ 0.50). The primary analysis will be undertaken by excluding patients with missing data. However, we will conduct sensitivity analysis for missing data using the following imputation techniques, as appropriate: (i) last value carried forward (LVCF); (ii) baseline value carried forward (BVCF); (iii) single imputation with EM algorithm; and (iv) multiple imputation. Data will be analysed using appropriate statistical software (eg SPSS, R, STATA).

Assuring instrument reliability

Clinical instruments requiring ratings by research staff will continue to be tested for inter-rater reliability across the two sites, SCARF and AIIMS. A joint meeting of the research staff from both sites was held and inter-rater reliability exercises on the important measures were conducted using video-recorded interviews with 10 patients with FEP. Data was successfully collected for the following measures: Scale for Assessment of Positive Symptoms, Scale for The Assessment of Negative Symptoms, Young Mania Rating Scale, Montgomery-Asberg Depression Rating Scale, Brief Psychiatric Rating Scale, Global Assessment of Functioning, Social and Occupational Functioning Assessment Scale and Medication Adherence Rating Scale. A high degree of inter-rater reliability across sites (intraclass correlations from 0.74 to 0.92) was obtained on these measures.

Ethics and dissemination

Ethics

Written, informed consent will be obtained from all participants or carers as appropriate, before their enrolment into the study. In the school and college-based study (WP-5), all participants will receive information on where to seek help for mental health problems. In addition, we have developed a clinical care pathway (Appendix I) for those who are identified as being at 'high-risk for severe mental illness or having undetected /untreated severe mental illness or with suicidal ideations that will ensure safety, protection of confidentiality and privacy. The study principal investigator and team will preserve the confidentiality and anonymity of participants taking part in the study and fulfil transparency requirements under the General Data Protection Regulation for health and care research. The study may be subject to inspection and audit by the University of Warwick under their remit as sponsor and other regulatory

For peer review only - http://bmjopen.bmj.com/site/about/guidelines.xhtml

bodies to make sure adherence to GCP and the UK Policy Framework for Health and Social Care Research.

This study has been approved by the University of Warwick's Biomedical and Scientific Research Ethics Committee (reference: REGO-2018-2208), Coventry, UK and research ethics committees of all participating organisations. The ethical conduct of the study is monitored throughout by the University of Warwick and each participating organisation.

Dissemination Strategies

We will strategically exploit our strong network with various LMIC stakeholders involved in mental health services such as clinicians, academics, service providers, policy makers, government and non-governmental agencies, community groups, patient and carer advocacy groups, media agencies and the creative arts industry to support WIC dissemination activities. We will use a variety of methods to ensure effective dissemination of the groups' work including; (a): development of an up-to-date project website (to target different audiences/stakeholders) (b) presentations at national and international psychiatric and mental health scientific conferences; peer review publications, and (c) publication through visual media including film and theatre. Dissemination activities through cinema and theatre have a particular resonance for many LMIC populations and we believe that we will reach a large number of people through these activities. We will work closely with our LMIC partners to identify the dissemination approaches that might work best in this setting.

Patient and Public Involvement

We have strong and ongoing engagement with various internal and external stakeholders including patients and public involved in mental health care, throughout all phases of research. The recommendations from focus group discussions with various stakeholders were invaluable, especially in understanding the patients and carers priorities, needs and experience. These insights were instrumental in the preparatory phase of WIC, including development of a bespoke study protocol for the clinical management of patients with psychotic disorders and development of interventions that can improve their access to clinical care. The patients and/or public were not involved in proposing the research objectives nor involved in the overall study design or the implementation of this research programme. However, local dissemination plans will be devised by the research team at respective sites with the various stakeholders involved in mental health.

For peer review only - http://bmjopen.bmj.com/site/about/guidelines.xhtml

Conclusion

Although major improvements have been achieved in the treatment of patients with psychotic disorders in high-income countries including the widescale implementation of EIS in psychosis, implementing such services remains a major challenge in LMICs. The sizeable differences in patient profiles, socio-demographics, pathways of care, barriers to care,comorbidities, scarcity of resources and delayed presentation means that Western models of care cannot be simply translocated to LMICs. The WIC programme will tailor evidence-informed interventions to the Indian socio-cultural context to address the increasing burden of psychotic disorders in India. By using a bespoke interventional approach that is regionally relevant, and by exploiting the untapped potential of digital and mobile technologies in India, WIC will develop and test interventions that can be integrated into existing clinical care, and generate data (including health economic data) that will inform priorities for healthcare providers and policy makers. ,

Disclaimer: This research was commissioned by the National Institute of Health Research using Official Development Assistance (ODA) funding. The views expressed are those of the author(s) and not necessarily those of the NIHR or the Department of Health and Social Care. [illegible]

Funding: United Kingdom's National Institute of Health Research (NIHR) using Official Development Assistance (ODA) funding (Award number: 16/137/107). [illegible]

Author Contributions: SPS is the Director of this global health research group. SPS conceived the original study design and obtained funding. MM is the programme manager and prepared the first draft and subsequent revisions of the manuscript, and is the joint first author with SPS. TR,PR, MS, JM, GC and RKC contributed writing to particular sections relevant to their work package of the Group. All other co-investigators provided critical comments during the proposal-writing stage, WIC project meetings and critically reviewed and revised the manuscript. All authors read and approved the final manuscript.

Conflicts of Interests

None

Data Statement

Data will be stored in University of Warwick repository for at least 10 years after its last access, in accordance with Research Data Management Policy, and will be made available to other users under data-sharing agreement. The decision on whether to

For peer review only - http://bmjopen.bmj.com/site/about/guidelines.xhtml

For peer review only

share research samples and/or supply data to a potential new user will be made by the Programme Management Group (PMG). All data requests should be submitted to the chief investigator for consideration.

Acknowledgements

We thank the WIC independent external scientific advisory group (ESAG), for providing strategic insight and international perspectives for WIC and also for their valuable inputs to the critical decisions affecting the scientific orientation of the WIC research programme. S.P.S and M.B are supported by the NIHR Applied Research Centre (ARC) West Midlands. S.N.I is supported by a salary award from the Canadian Institutes of Health Research.

For peer review only

For peer review only - http://bmjopen.bmj.com/site/about/guidelines.xhtml

References

1. Collaborators GDaIIaP. Global, regional, and national incidence, prevalence, and years lived with disability for 354 diseases and injuries for 195 countries and territories, 1990-2017: a systematic analysis for the Global Burden of Disease Study 2017. *Lancet* 2018;392(10159):1789-858. doi: 10.1016/S0140-6736(18)32279-7 [published Online First: 2018/11/08]
2. Farooq S. Early intervention for psychosis in low- and middle-income countries needs a public health approach. *Br J Psychiatry* 2013;202(3):168-9. doi: 10.1192/bjp.bp.112.113761
3. Rathod S, Pinninti N, Irfan M, et al. Mental Health Service Provision in Low- and Middle-Income Countries. *Health Serv Insights* 2017;10:1178632917694350. doi: 10.1177/1178632917694350 [published Online First: 2017/03/28]
4. Shiers D, Lester H. Early intervention for first episode psychosis. *BMJ* 2004;328(7454):1451-2. doi: 10.1136/bmj.328.7454.1451 [published Online First: 2004/06/19]
5. Sudharsanan N BD. The Demography of Aging in Low- and Middle-Income Countries: Chronological versus Functional Perspectives. . *M Majumdar, M Hayward, M Majumdar, M Hayward (Eds), Future directions for the demography of aging (1st ed), National Academies Press, Washington, DC* 2018:pp. 309-38.
6. Chatterjee S, Pillai A, Jain S, et al. Outcomes of people with psychotic disorders in a community-based rehabilitation programme in rural India. *Br J Psychiatry* 2009;195(5):433-9. doi: 10.1192/bjp.bp.108.057596
7. Malla A, McGorry P. Early Intervention in Psychosis in Young People: A Population and Public Health Perspective. *Am J Public Health* 2019;109(S3):S181-S84. doi: 10.2105/AJPH.2019.305018 [published Online First: 2019/06/27]
8. Correll CU, Galling B, Pawar A, et al. Comparison of Early Intervention Services vs Treatment as Usual for Early-Phase Psychosis: A Systematic Review, Meta-analysis, and Meta-regression. *JAMA Psychiatry* 2018;75(6):555-65. doi: 10.1001/jamapsychiatry.2018.0623 [published Online First: 2018/05/26]
9. Chiliza B, Asmal L, Emsley R. Early intervention in schizophrenia in developing countries: focus on duration of untreated psychosis and remission as a treatment goal. *Int Rev Psychiatry* 2012;24(5):483-8. doi: 10.3109/09540261.2012.704873
10. McGorry PD, Ratheesh A, O'Donoghue B. Early Intervention-An Implementation Challenge for 21st Century Mental Health Care. *JAMA Psychiatry* 2018;75(6):545-46. doi: 10.1001/jamapsychiatry.2018.0621
11. Singh SP. Early intervention in psychosis. *Br J Psychiatry* 2010;196(5):343-5. doi: 10.1192/bjp.bp.109.075804
12. McGorry PD, Hartmann JA, Spooner R, et al. Beyond the "at risk mental state" concept: transitioning to transdiagnostic psychiatry. *World Psychiatry* 2018;17(2):133-42. doi: 10.1002/wps.20514
13. Chokshi M, Patil B, Khanna R, et al. Health systems in India. *J Perinatol* 2016;36(s3):S9-S12. doi: 10.1038/jp.2016.184
14. Lamers LM, McDonnell J, Stalmeier PF, et al. The Dutch tariff: results and arguments for an effective design for national EQ-5D valuation studies. *Health Econ* 2006;15(10):1121-32. doi: 10.1002/hec.1124

For peer review only

15. Singh SP. Early intervention in the Indian context: In S. Malhotra & S. Chakrabarti (Eds.) 2015:89-98.
16. Large M, Farooq S, Nielssen O, et al. Relationship between gross domestic product and duration of untreated psychosis in low- and middle-income countries. *Br J Psychiatry* 2008;193(4):272-8. doi: 10.1192/bjp.bp.107.041863
17. Chadda RK, Agarwal V, Singh MC, et al. Help seeking behaviour of psychiatric patients before seeking care at a mental hospital. *Int J Soc Psychiatry* 2001;47(4):71-8. doi: 10.1177/002076400104700406
18. Hanna F, Barbui C, Dua T, et al. Global mental health: how are we doing? *World Psychiatry* 2018;17(3):367-68. doi: 10.1002/wps.20572
19. Garg K, Kumar CN, Chandra PS. Number of psychiatrists in India: Baby steps forward, but a long way to go. *Indian J Psychiatry* 2019;61(1):104-05. doi: 10.4103/psychiatry.IndianJPsychiatry_7_18
20. Gururaj G VM, Benegal V, Rao GN, Pathak K, Singh LK, Mehta RY, Ram D,, Shibukumar TM KA, Lenin Singh RK, Chavan BS, Sharma P, Ramasubramanian, C DP, Saha PK, Deuri SP, Giri AK, Kavishvar AB, Sinha VK, Thavody J, Chatterji R,, et al. National Mental Health Survey of India, 2015-16: Summary. Bengaluru, National
Institute of Mental Health and Neuro Sciences. Bengaluru: NIMHANS, 2016.
21. Saxena S, Sharan P. [Supporting mental health research publications from low- and middle-income countries]. *Braz J Psychiatry* 2004;26(2):73-4. doi: 10.1590/s1516-44462004000200001 [published Online First: 2004/11/02]
22. Chadda RK. Caring for the family caregivers of persons with mental illness. *Indian J Psychiatry* 2014;56(3):221-7. doi: 10.4103/0019-5545.140616 [published Online First: 2014/10/16]
23. Koschorke M, Padmavati R, Kumar S, et al. Experiences of stigma and discrimination of people with schizophrenia in India. *Soc Sci Med* 2014;123:149-59. doi: 10.1016/j.socscimed.2014.10.035 [published Online First: 2014/10/18]
24. Keshavan MS, Shrivastava A, Gangadhar BN. Early intervention in psychotic disorders: Challenges and relevance in the Indian context. *Indian J Psychiatry* 2010;52(Suppl 1):S153-8. doi: 10.4103/0019-5545.69228
25. Birchwood M, Lester H, McCarthy L, et al. The UK national evaluation of the development and impact of Early Intervention Services (the National EDEN studies): study rationale, design and baseline characteristics. *Early Interv Psychiatry* 2014;8(1):59-67. doi: 10.1111/eip.12007 [published Online First: 2013/01/26]
26. Singh SP, Islam Z, Brown LJ, et al. Ethnicity, detention and early intervention: reducing inequalities and improving outcomes for black and minority ethnic patients: the ENRICH programme, a mixed-methods study2013.
27. Singh SP, Brown L, Winsper C, et al. Ethnicity and pathways to care during first episode psychosis: the role of cultural illness attributions. *BMC Psychiatry* 2015;15:287. doi: 10.1186/s12888-015-0665-9 [published Online First: 2015/11/16]
28. Singh SP, Cooper JE, Fisher HL, et al. Determining the chronology and components of psychosis onset: The Nottingham Onset Schedule (NOS). *Schizophr Res* 2005;80(1):117-30. doi: 10.1016/j.schres.2005.04.018 [published Online First: 2005/06/22]
29. van Ginneken N, Jain S, Patel V, et al. The development of mental health services within primary care in India: learning from oral history. *Int J Ment Health Syst* 2014;8:30. doi: 10.1186/1752-4458-8-30 [published Online First: 2014/07/16]

For peer review only - http://bmjopen.bmj.com/site/about/guidelines.xhtml

30. Shidhaye R. Implementation Science for closing the treatment gap for mental disorders by translating evidence base into practice: experiences from the PRIME project. *Australas Psychiatry* 2015;23(6 Suppl):35-7. doi: 10.1177/1039856215609771
31. Yapa HM, Bärnighausen T. Implementation science in resource-poor countries and communities. *Implement Sci* 2018;13(1):154. doi: 10.1186/s13012-018-0847-1 [published Online First: 2018/12/27]

For peer review only

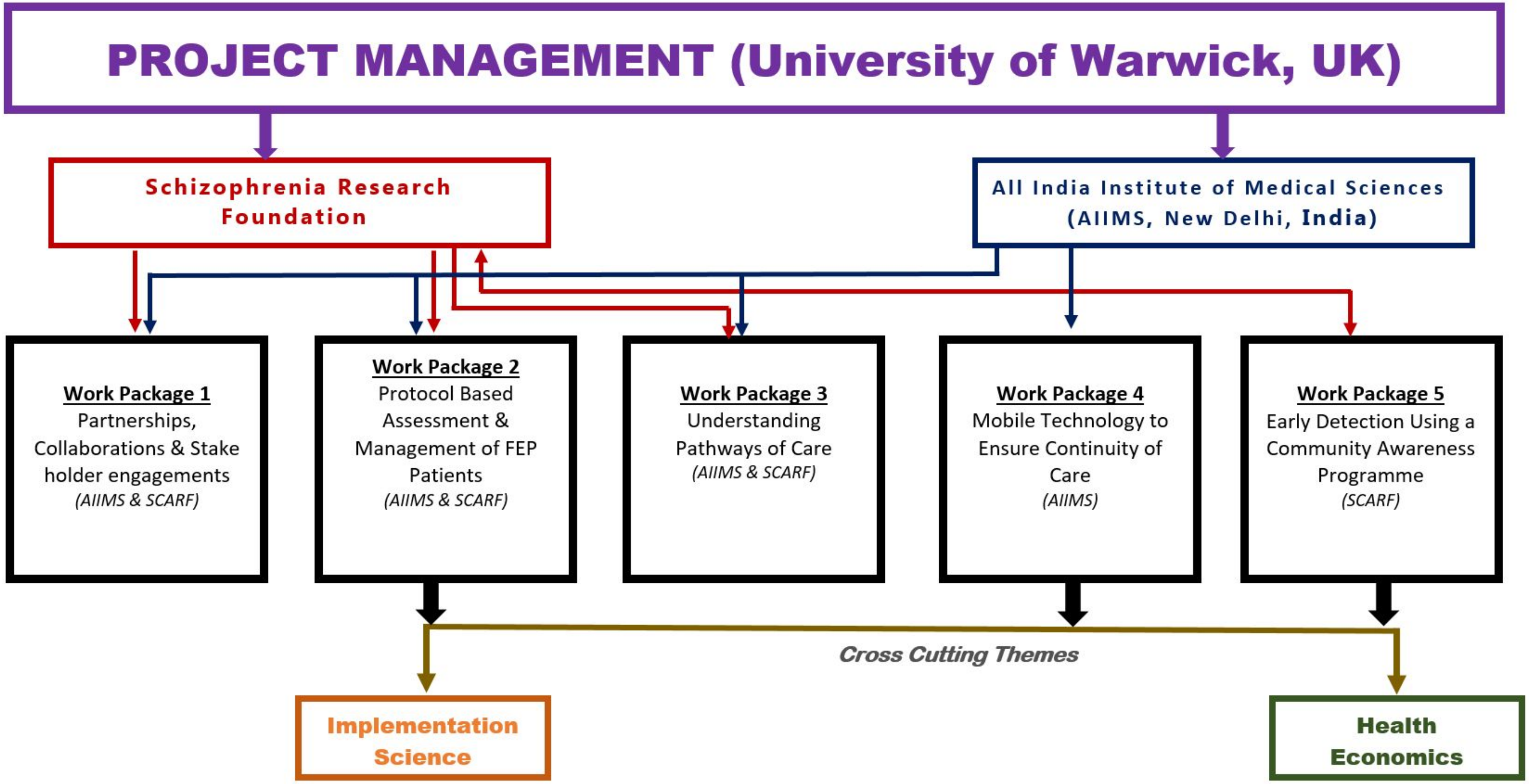

Figure 1: Schematic Overview of WIC GHRG. WP, Work Package

For peer review only

For peer review only - http://bmjopen.bmj.com/site/about/guidelines.xhtml

For peer review only

For peer review only - http://bmjopen.bmj.com/site/about/guidelines.xhtml

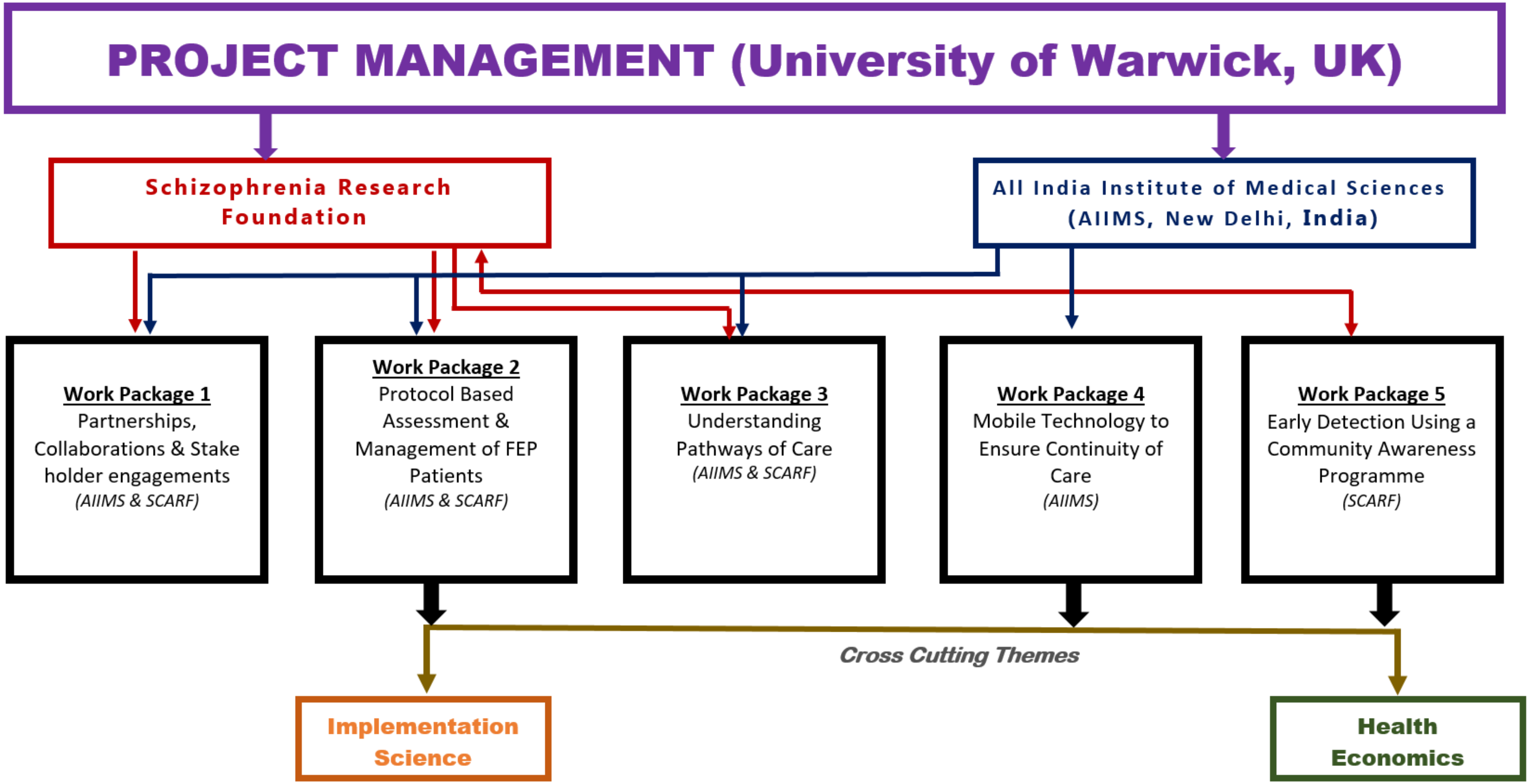

Figure 1: Schematic Overview of WIC GHRG. WP, Work Package

For peer review only

Section A – *Work Package 2:- Protocol-based assessment and management of FEP*

Study Population

Patients with 'untreated' FEP and their carers visiting psychiatry department at respective collaborating sites, fulfilling the study inclusion/exclusion criteria will be recruited into the study. Participating patients must be aged 16–45 years with a diagnosis of FEP based on the International Classification of Diseases, 10th revision (ICD-10) codes for schizophrenia (F20), persistent delusional disorder (F22), acute and transient psychotic disorders (F 23), schizoaffective disorder (F25), other and unspecified psychosis (F28-29), mania with psychotic symptoms (F30.2), bipolar affective disorder, current episode manic with psychotic symptoms (F 31.2), severe depressive episode with psychotic symptoms (F32.3) and recurrent depressive disorder and/or current episode depression with psychotic symptoms (F 33.3). Untreated is defined as not having received anti-psychotic medication for more than 30 days since the onset of psychosis. Patients must be able to communicate in Tamil or English in Chennai, and Hindi or English in New Delhi. Study exclusion criteria are: those with overt learning disability (equivalent to IQ <70), organic brain damage and epilepsy of pervasive developmental disorder. Written informed consent will be obtained from interested and eligible participants prior to participation. Consent will also be sought from interested carers of study participants for carer-reported measures.

Study Assessment Tools

Various clinical scales will be used to assess several domains of symptom severity and patient functioning at baseline and follow-up assessments. Baseline assessment will be conducted soon after entry into the service, and follow-up assessments will be conducted at 3, 6 and 12 months.

Socio-demographic, clinical, and socio/family variables will be collected using semi-structured (MRC demographic schedule) face-to-face clinical interview. Psychopathology will be assessed using the: (i) Scale for the Assessment of Positive Symptoms (SAPS) (1); (ii) Scale for the Assessment of Negative Symptoms (SANS) (1); (iii) Young Mania Rating Scale (YMRS) (2); (iv) Montgomery - Asberg Depression Rating Scale (3); and (v) Brief Psychiatric Rating Scale (BPRS) (4). Severity of illness and functioning will be measured using the Global Assessment of Functioning (GAF) Scale (5), the Social And Occupational Functioning Assessment Scale (SOFAS) (6)

For peer review only

and 5-level EQ-5D (EQ 5 D 5L) (7). Other assessment tools that will be used in this study are: (i) The Emerging Psychosis Attribution Schedule (EPAS) to assess patient and carer attributions of symptoms in the emerging phase of a psychotic episode (8); (ii) the Nottingham Onset Schedule (NOS) to establish duration of untreated psychosis (9); (iii) an amended version of the Encounter form to assess service encounters during the psychotic phase of the illness (8); (iv) Medication Adherence Rating Scale (MARS) for assessment of medication adherence in psychosis(10); (v) an assessment schedule to assess the nature of insight in psychosis (11); (vi) tools for assessment of risk factors (trauma exposure) including the Childhood Trauma Questionnaire (CTQ) (12), Harvard Trauma Questionnaire (HTQ) (13) and the List Of Threatening Experiences (LTE) (14); and (vii) Brief Assessment of Cognition in Schizophrenia (BACS) for neurocognition. Additionally, we will also capture data on treatment, relapse and physical health. The caregivers will be assessed on the Burden Assessment Schedule (BAS) (15). Engagement of caregivers will be encouraged to obtain accurate patient-information including patients' medical history, risk factors, family responses to illness, help-seeking and potential impact on family. Table 1 depicts the assessment schedule for both baseline and follow-up assessments.

For peer review only

Table 1: Study Assessment Schedule of WIC

Study Instruments	Baseline	Month 3	Month 6	Month 12
MRC Sociodemographic Schedule	X	-	-	-
Nottingham Onset Schedule (NOS)	X	-	-	-
Emerging Psychosis Attribution Schedule (EPAS)	X	-	-	-
Service Encounters Interview	X	-	-	-
Scale for Assessment of Positive Symptoms (SAPS)	X	X	X	X
Scale for Assessment of Negative Symptoms (SANS)	X	X	X	X
Young Mania Rating Scale (YMRS)	X	X	X	X
Montgomery-Asberg Depression Rating Scale	X	X	X	X
Brief Assessment of Cognition in Schizophrenia (BACS)	X	X	X	X
Global Assessment of Functioning(GAF)	X	X	X	X
Social and Occupational Functioning Assessment Scale (SOFAS)	X	X	X	X
Brief Psychiatric Rating Scale (BPRS)	X	X	X	X
Medication Adherence Rating Scale (MARS)	X	X	X	X
EQ-5D 5 L	X	X	X	X
Burden Assessment Schedule (BAS) – Carer	X	X	X	X
Risk Factor Assessment (CTQ, HTQ & LTE)	X	-	-	-
Physical Health Assessment, treatment, relapse*	X	X	X	X
Analysis of Cost*	X	X	X	X

**Investigator designed ; CTQ, childhood trauma questionnaire; HTQ, Harvard trauma questionnaire; LTE, list of threatening experiences (LTE)*

For peer review only

Section B – <u>Work Package 4:- Mobile technology to ensure continuity of care (Commissioned Project)</u>

Background

In India, over 90% of patients stay in the community with families, and in the absence of state provision, families initiate treatment, procure and supervise medicines and provide psychosocial support including opportunities for education, vocation and recreation (16). Importantly, the stress of caregiving adversely affects carer's' physical and mental health(17). Digital technology platforms have tremendous potential to effectively facilitate continuity of clinical care for mental illnesses, where state sponsored provisions are scarce, such as India. Globally, India has the second largest number of mobile phone users – 900 million users accounting for 88% of all telecommunication users (18). Studies have emerged from high income countries on mobile technologies in psychosis (19) but are lacking in LMICs (20). Our survey shows that 88% of patients have mobile phones (21). In this WP, we will deliver a home-based psycho-social recovery care model, strategies for reducing carer burden, and evaluate its clinical and cost-effectiveness using a three-pronged approach (Figure 2). In brief, first, we will conduct focus group discussions with patients with schizophrenia, their families/caregivers and mental health professionals, to gather information on their healthcare needs and expected outcomes from a home-based psycho-social care model. Second, utilizing insights from these focus groups and an extensive literature review, we will develop a 'bespoke' home-based psycho-social care model (named as SAKSHAM, meaning effort in Hindi, booklet version) aimed at improving functioning and recovery of 'difficult-to-treat' patients with schizophrenia; and at reducing carer burden. Third, we will develop a mobile application based on the SAKSHAM booklet. Finally, we will evaluate the feasibility and effectiveness of both the booklet version and mobile based application in a small group of patients with schizophrenia and their caregivers, and will compare against treatment as usual group. This study will be conducted at AIIMS, New Delhi, India. For an overview of study inclusion/exclusion criteria of study cohort and list of study instruments with assessment schedule, see Table 2 and Table 3, respectively.

For peer review only

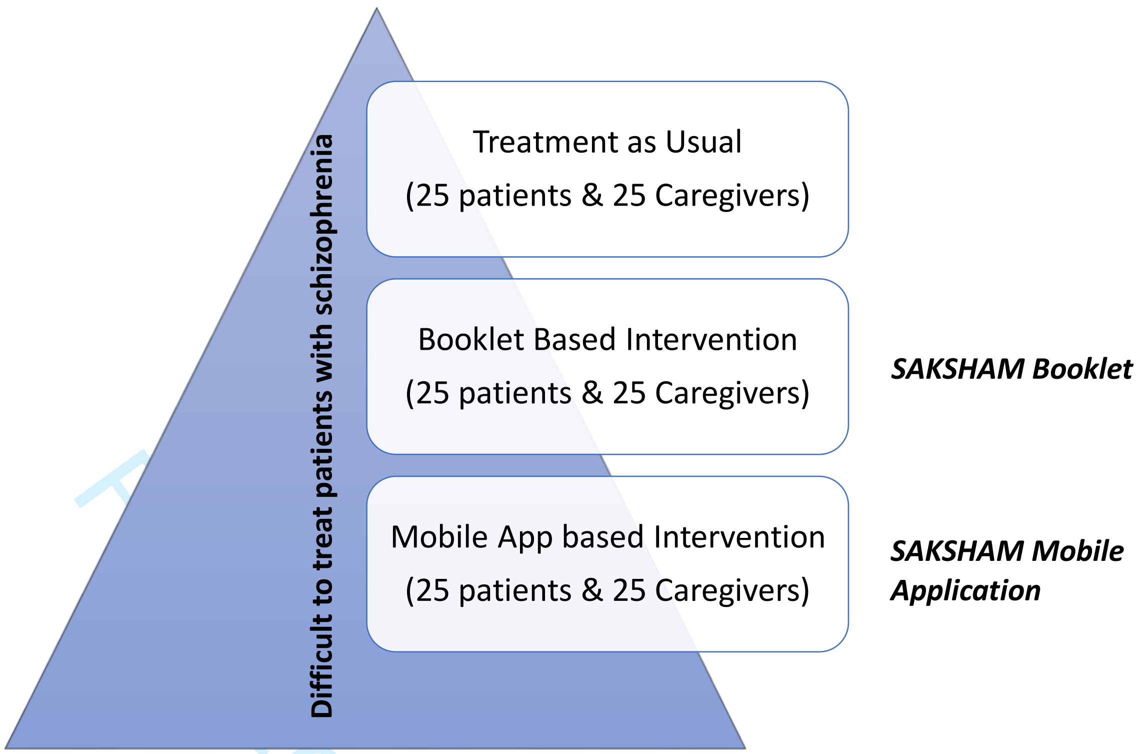

Figure 2 : Three arm study to evaluate the feasibility and effectiveness of home-based psycho-social recovery care model

For peer review only

Table 2 : Study Inclusion /Exclusion Criteria for Patients and their Caregivers.

Patients	Caregivers
Inclusion Criteria	***Inclusion Criteria***
Aged 18–65 years	Immediate family member fulfilling any three of following criteria:<br>• Spending maximum time with the patient.<br>• Supporting the patient financially<br>• Most involved in the care of the patient<br>• Able to be contacted by treatment staff in case of an emergency
Diagnosis of schizophrenia based on International Classification of Diseases - Tenth Revision (ICD-10)codes [Schizophrenia (F20), Persistent delusional disorder (F22), Schizoaffective disorder (F25), Other and Unspecified Psychosis (F28 & F29)]	Using mobile device for at least one year
Duration of illness ≥ 2 years	Able to read Hindi or English
Persistent significant psychopathology defined as score of ≥4 (moderate) on items P1-P7 and items N1-N7 items 7, 20, 25 or 34 on Positive and Negative Syndrome Scale (PANSS).	***Exclusion Criteria***
Persistent significant psychopathology and dysfunction, despite receiving regular treatment	Caregivers suffering from psychiatric disorder which might interfere with the care of patients and his/her co-operation during
Using mobile device for at least one year	Not willing to provide informed consent
Able to read Hindi or English	
Exclusion Criteria	
Unable to provide informed consent	

For peer review only

Table 3 : Study Assessment Schedule for WP 4

Study Instruments	Baseline	Month 1	Month 3
**Assessment of Patients**			
Socio-demographic & Clinical Characteristics	X	X	X
Assessment of Psychopathology:	X	X	X
Positive and Negative Syndrome Scale	X	X	X
Schizophrenia Cognitive Rating Scale (SCoRS)	X	X	X
Assessment of Physical Health	X	X	X
Assessment of Treatment Adherence:	X	X	X
Medication Adherence Rating Scale (MARS)	X	X	X
Assessment of Functioning:	X	X	X
Katz Index of Independence in Activities of Daily Living (ADL)	X	X	X
Lawton Brody Instrumental Activities of Daily Living (IADL)	X	X	X
World Health Organization Disability Assessment Schedule (WHODAS)2.0	X	X	X
Clinical Global Improvement	X	X	X
Assessment of Caregivers			
Mini International Neuropsychiatric Interview (MINI) Screen for caregivers	X	X	X
Family Burden Interview Schedule	X	X	X
Ways of Coping Checklist-Hindi Adaptation (WCC-HA)	X	X	X

For peer review only

Section C – Work Package 5:-Early detection using a community awareness programme (Commissioned Project)

India has a young population – over half of Indian's population are under the age of 30 years (22), with an overall mental illness prevalence rate of 10.6% and nearly 150 million Indians in need of active interventions (23). Taking into account that 80% of FEP usually occurs at a very young age (i.e. between 16 and 30 years of age) (24) and that LMICs have predominantly young populations, it is highly likely that an overwhelming majority of people experiencing FEP live in LMIC countries such as India. However, lack of adequate mental health services in India along with other impediments to care such as stigma and lack of awareness can lead to delayed help-seeking contributing to long DUPs. A longer duration of DUP is associated with a poor treatment response, greater subsequent disability and higher mortality rates (25); therefore, timely identification and diagnosis are pivotal. India does not have standardized early detection and intervention programs for psychotic disorders. There are sporadic awareness and screening programs held in some schools and colleges. Moreover, these programs have not integrated active referral of "cases" to appropriate interventions. This work package therefore aims to increase mental health literacy of teachers and students; to use a web-based screening tool to detect youth who could benefit from mental health services; and to refer such youth to appropriate services.

Table 4 : List of study instruments

**Knowledge, Attitude and Practice (KAP) questionnaires**
The Mental Health Knowledge Schedule<br>The Reported and Intended Behaviour scale
**Mental health screening tool**
Patient Health Questionnaire-9 (PHQ-9)<br>Generalized Anxiety Disorder-7 scale (GAD-7)<br>Community Assessment of Psychic Experiences – Positive 15-items Scale (CAPE-P15)

Intervention

We will develop mental health training modules for teachers and mental health awareness modules for students. Data on Knowledge, Attitude and Practice (KAP) of

For peer review only

the teachers and students will be obtained at baseline, month 12 and month 24. Training for teachers include one half day session (4 hours) and three subsequent booster sessions (1 hour) in three-month intervals. Students will be provided education on mental health – one half day session (4 hours). Voluntary mental health screening tool with the PHQ-9, GAD-7 and CAPE-P15 will be made available to all students through web-based applications and mobile applications.

References

1. Andreasen NC, Arndt S, Miller D, Flaum M, Nopoulos P. Correlational studies of the Scale for the Assessment of Negative Symptoms and the Scale for the Assessment of Positive Symptoms: an overview and update. Psychopathology. 1995;28(1):7-17.
2. Young RC, Biggs JT, Ziegler VE, Meyer DA. A rating scale for mania: reliability, validity and sensitivity. Br J Psychiatry. 1978;133:429-35.
3. Montgomery SA, Asberg M. A new depression scale designed to be sensitive to change. Br J Psychiatry. 1979;134:382-9.
4. Overall JE, Gorham DR. The Brief Psychiatric Rating Scale. Psychological Reports. 1962;10(3):799-812.
5. Jones SH, Thornicroft G, Coffey M, Dunn G. A brief mental health outcome scale-reliability and validity of the Global Assessment of Functioning (GAF). Br J Psychiatry. 1995;166(5):654-9.
6. Skodol AE, Link BG, Shrout PE, Horwath E. Toward construct validity for DSM-III Axis V. Psychiatry Res. 1988;24(1):13-23.
7. Herdman M, Gudex C, Lloyd A, Janssen M, Kind P, Parkin D, et al. Development and preliminary testing of the new five-level version of EQ-5D (EQ-5D-5L). Qual Life Res. 2011;20(10):1727-36.
8. Singh SP, Brown L, Winsper C, Gajwani R, Islam Z, Jasani R, et al. Ethnicity and pathways to care during first episode psychosis: the role of cultural illness attributions. BMC Psychiatry. 2015;15:287.
9. Singh SP, Cooper JE, Fisher HL, Tarrant CJ, Lloyd T, Banjo J, et al. Determining the chronology and components of psychosis onset: The Nottingham Onset Schedule (NOS). Schizophr Res. 2005;80(1):117-30.
10. Vanelli I, Chendo I, Gois C, Santos J, Levy P. [Medication adherence rating scale]. Acta Med Port. 2011;24(1):17-20.
11. David A, Buchanan A, Reed A, Almeida O. The assessment of insight in psychosis. Br J Psychiatry. 1992;161:599-602.
12. Pennebaker JW, Susman JR. Disclosure of traumas and psychosomatic processes. Soc Sci Med. 1988;26(3):327-32.
13. Mollica RF, Caspi-Yavin Y, Bollini P, Truong T, Tor S, Lavelle J. The Harvard Trauma Questionnaire. Validating a cross-cultural instrument for measuring torture, trauma, and posttraumatic stress disorder in Indochinese refugees. J Nerv Ment Dis. 1992;180(2):111-6.
14. Brugha TS, Cragg D. The List of Threatening Experiences: the reliability and validity of a brief life events questionnaire. Acta Psychiatr Scand. 1990;82(1):77-81.
15. Thara R, Padmavati R, Kumar S, Srinivasan L. Instrument to assess burden on caregivers of chronic mentally ill. Indian J Psychiatry. 1998;40(1):21-9.

For peer review only

16. Chadda RK. Caring for the family caregivers of persons with mental illness. Indian J Psychiatry. 2014;56(3):221-7.
17. Chadda RK, Singh TB, Ganguly KK. Caregiver burden and coping: a prospective study of relationship between burden and coping in caregivers of patients with schizophrenia and bipolar affective disorder. Soc Psychiatry Psychiatr Epidemiol. 2007;42(11):923-30.
18. Stalin P, Abraham SB, Kanimozhy K, Prasad RV, Singh Z, Purty AJ. Mobile Phone Usage and its Health Effects Among Adults in a Semi-Urban Area of Southern India. J Clin Diagn Res. 2016;10(1):LC14-6.
19. Firth J, Torous J. Smartphone Apps for Schizophrenia: A Systematic Review. JMIR Mhealth Uhealth. 2015;3(4):e102.
20. Sood M, Chadda RK, Singh P. Mobile health (mHealth) in mental health: Scope and applications in low-resource settings. Natl Med J India. 2016;29(6):341-3.
21. M Sood RC, K Sinha Deb, R Bhad , A Mahapatra, R Verma, AK Mishra. Scope of Mobile Phones in Mental Health Care in Low Resource Settings. Journal of Mobile Technology in Medicine. 2016(5:2:33–37).
22. Factbook CIAW. India Demographics Profile 2018 2018 [updated January 20 2018. Available from: https://www.indexmundi.com/india/demographics_profile.html.
23. Murthy RS. National Mental Health Survey of India 2015-2016. Indian J Psychiatry. 2017;59(1):21-6.
24. Shiers D, Lester H. Early intervention for first episode psychosis. BMJ. 2004;328(7454):1451-2.
25. Farooq S, Large M, Nielssen O, Waheed W. The relationship between the duration of untreated psychosis and outcome in low-and-middle income countries: a systematic review and meta analysis. Schizophr Res. 2009;109(1-3):15-23.

For peer review only

BMJ Open

The Warwick-India-Canada (WIC) Global Mental Health Group: Rationale, Design and Protocol.

Journal:	*BMJ Open*
Manuscript ID	bmjopen-2020-046362.R1
Article Type:	Protocol
Date Submitted by the Author:	08-Mar-2021

Complete List of Authors:	Singh, Swaran; University of Warwick Warwick Medical School, Division of Mental Health & Wellbeing<br>Mohan, Mohapradeep; University of Warwick Warwick Medical School,<br>Iyer, Srividya ; McGill University, Douglas Mental Health University Institute<br>Meyer, Caroline; University of Warwick,<br>Currie, Graeme; Warwick Business School<br>Shah, Jai; McGill University, Department of Psychiatry, McGill University<br>Madan, Jason; University of Warwick Warwick Medical School<br>Birchwood, Max; University of Warwick, Division of mental health & well-being<br>Sood, Mamta; All India Institute of Medical Sciences, Department of Psychiatry<br>Padmavati, Ramachandran; Schizophrenia Research Foundation<br>Chadda, Rakesh; All India Institute of Medical Sciences, Psychiatry<br>Lilford, RJ; University of Birmingham,<br>Thara, Rangaswamy; SCARF<br>Furtado, Vivek; University of Warwick Warwick Medical School, Mental Health and Wellbeing; Birmingham and Solihull Mental Health NHS Foundation Trust, Forensic Psychiatry

**Primary Subject Heading**:	Mental health
Secondary Subject Heading:	Health economics, Health services research, Mental health, Qualitative research
Keywords:	Schizophrenia & psychotic disorders < PSYCHIATRY, Child & adolescent psychiatry < PSYCHIATRY, Adult psychiatry < PSYCHIATRY

SCHOLARONE™
Manuscripts

I, the Submitting Author has the right to grant and does grant on behalf of all authors of the Work (as defined in the below author licence), an exclusive licence and/or a non-exclusive licence for contributions from authors who are: i) UK Crown employees; ii) where BMJ has agreed a CC-BY licence shall apply, and/or iii) in accordance with the terms applicable for US Federal Government officers or employees acting as part of their official duties; on a worldwide, perpetual, irrevocable, royalty-free basis to BMJ Publishing Group Ltd ("BMJ") its licensees and where the relevant Journal is co-owned by BMJ to the co-owners of the Journal, to publish the Work in this journal and any other BMJ products and to exploit all rights, as set out in our licence.

The Submitting Author accepts and understands that any supply made under these terms is made by BMJ to the Submitting Author unless you are acting as an employee on behalf of your employer or a postgraduate student of an affiliated institution which is paying any applicable article publishing charge ("APC") for Open Access articles. Where the Submitting Author wishes to make the Work available on an Open Access basis (and intends to pay the relevant APC), the terms of reuse of such Open Access shall be governed by a Creative Commons licence – details of these licences and which Creative Commons licence will apply to this Work are set out in our licence referred to above.

Other than as permitted in any relevant BMJ Author's Self Archiving Policies, I confirm this Work has not been accepted for publication elsewhere, is not being considered for publication elsewhere and does not duplicate material already published. I confirm all authors consent to publication of this Work and authorise the granting of this licence.

For peer review only

The Warwick-India-Canada (WIC) Global Mental Health Group: Rationale, Design and Protocol.

Swaran Preet Singh*,[1] Mohapradeep Mohan*,[1] Srividya N Iyer, [2] Caroline Meyer,[3] Graeme Currie,[4] Jai Shah,[2] Jason Madan,[5] Max Birchwood,[1] Mamta Sood,[6]; Padmavati Ramachandran,[7] Rakesh K Chadda, [6] Richard J Lilford,[8] Thara Rangaswamy, [7] Vivek Furtado, [1] and on behalf of the WIC Consoritum

** S.P.S and MM contributed equally*

Author Affiliations

(1) Division of Mental Health and Wellbeing, Warwick Medical School, University of Warwick, Coventry, UK

(2) Douglas Mental Health University Institute, McGill University, Montreal, Canada

(3) WMG and Warwick Medical School, University of Warwick, Coventry, UK

(4) Warwick Business School, University of Warwick, Coventry, UK

(5) Warwick Clinical Trials Unit, Warwick Medical School, University of Warwick, Coventry, UK

(6) Department of Psychiatry, All India Institute of Medical Sciences, rg, Ansari Nagar,, New Delhi, India.

(7) Schizophrenia Research Foundation, R/7A, North Main Road, Anna Nagar West Extension, Chennai, Tamil Nadu, India

(8) Institute of Applied Health Research, University of Birmingham, Birmingham, UK.

Word Count : 4333

Corresponding Author

Prof Swaran Preet Singh, Division of Mental Health and Wellbeing, Warwick Medical School, University of Warwick, Coventry, UK.

Email: S.P.Singh@warwick.ac.uk

For peer review only

Abstract

Introduction

The primary aim of the NIHR funded global health research group, Warwick-India-Canda (WIC), is to reduce the burden of psychotic disorders in India. India has a large pool of undetected and untreated patients with psychosis and a treatment gap exceeding 75%. Evidence-based packages of care have been piloted, but delivery of treatments still remains a challenge. Even when patients access treatment, there is minimal to no continuity of care. The overarching ambition of WIC programme is to improve patient outcomes through: (i) developing culturally tailored clinical interventions ; (ii) early identification and timely treatment of individuals with mental illness; and (iii) improving access to care by exploiting the potential of digitial technologies.

Methods and Analysis

This multicentre, multi-component research programme, comprising five work packages (WPs) and two cross-cutting themes(CTs), is being conducted at two sites in India: Schizophrenia Research Foundation (SCARF), Chennai (South India) and All India Institute of Medical Sciences (AIIMS), New Delhi (North India). WIC will: (i) develop and evaluate evidence-informed interventions for early and first-episode psychosis; (ii) determine pathways of care for early psychosis; (iii) investigate the efficacy and cost-effectiveness of community care models, including digital and mobile technologies; (iv) develop strategies to reduce the burden of mental illnesses among youth; (v) assess the economic burden of psychosis on patients and their carers; and (vi) determine the feasibility of an early intervention in psychosis programme in India.

Ethics and Dissemination

This study has been approved by the University of Warwick's Biomedical and Scientific Research Ethics Committee (reference: REGO-2018-2208), Coventry, UK and research ethics committees of all participating organisations. Research findings will be disseminated through peer-reviewed scientific publications, presentations at learned societies, and visual media.

Keywords: First episode psychosis, Schizophrenia, Mental health, Low-and-middle-income countries, NIHR Global Health Research Group, WIC

Article Summary

Strengths and Limitations of this Study

- WIC will develop a bespoke clinical interventions with proven efficacy that is culturally adapted, appropriate, accessible and acceptable.
- This is the first multisite study to identify barriers, facilitators and economic evaluation for implementing early intervention services in psychosis in India.
- WIC will build research capacity with potential for future efforts, including in methodologies such as health economic evaluation and implementation science.
- WIC will identify pathways to mental health care in India and develop community-based models for continuity of clinical care.
- Some studies embedded are exploratory and hypothesis generating, therefore large studies may be warranted for conclusive evidence.

For peer review only

Introduction

Globally, psychotic disorders such as schizophrenia, are among the top 20 leading contributors to disability [1]. Nearly 80% of individuals develop their first episode of psychosis (FEP) during adolescence or early adulthood [2]. Importantly, the burden of disease and disability is particularly high in low-and-middle-income countries (LMICs) [3]. LMICs' have predominantly young populations, with the bulk of FEP cases receiving inadequate care [3-5]. Despite efforts to develop healthcare models for accessible and effective care in LMICs, the implementation of such models remains a major challenge [6].

In the developed world, among the most significant reforms since deinstitutionalisation, has been the establishment of early intervention in psychosis services (EIS) [7]. Given their clinical and cost-effectiveness [8-10], many countries have scaled up EIS, as part of their mental health policy. EIS focus on reducing the duration of untreated psychosis (DUP), provision of holistic and evidence-based specialist care during the 'critical period' of 2-5 years around onset of psychosis and interventions for individuals who are at high risk of developing a psychotic disorder (clinical high risk or ultra-high risk) [11 12]. Despite such promising evidence, many LMICs have not implemented EIS into their mental health systems. Inadequate mental health workforce, fragmented healthcare systems [13], and scarcity of research and implementation capacity are significant barriers to introducing such care in LMICs [14 15]. Furthermore, DUP is significantly higher in LMICs [16], and therefore EIS provision adapted to LMIC settings is potentially of great value. Help-seeking in LMICs is often pluralistic, with traditional practitioners being the initial, and sometimes the only source of care[17]. Therefore, a simple 'transplantation' of the 'Western' EIS model to LMIC context is neither feasible nor practical. However, it may be possible to incorporate the principles and therapeutic components of EIS into locally contextualised mental health care in LMICs, [3 18]. This is the key premise underpinning the WIC project.

Aim and Strategic Goals

The National Institute of Health Research (NIHR) Global Health Research Group (GHRG) on Psychosis Outcomes: the Warwick-India-Canada Network (WIC) aims to reduce the burden of psychotic disorders and improve health, wellbeing and functioning of those with psychotic disorders in India. The WIC network brings together knowledge and expertise of four internationally recognised institutions that share research interests, expertise in effective interventions in psychotic disorders, strategic

For peer review only

vision to reduce the burden of psychotic disorders in resource-poor settings, and who are keen to expand their research knowledge to implement evidence-based clinical changes in LMIC settings. Informed by principles of evidence-based EIS developed in the UK and Canada, the WIC GHRG will develop culturally sensitive interventions that will expand mental health services to communities that currently have limited access in India. WIC will also build and sustain much-needed research capacity and support the implementation of evidence-based practice.

The Indian Context

India with its 1.3 billion population, has a severe shortage of mental health care provision. Compared with a global average of 3.96 psychiatrists/ 100,000 population, India has 0.75 psychiatrists/100,000 population. The overall availability of mental healthcare workers (psychiatrists, clinical psycologists, and psychiatric social workers) is <1/ 100,000 population. [19 20]. India therefore has a large number of patients with undetected, untreated or inadequately treated patients with psychotic disorders [21]. Even when patients access care, there is no continuity; treatment is sporadic and focused on symptomatic control and crisis management rather than recovery [22]. Social stigma and discrimination associated with mental illness are also major impediments to delivering evidence-based care [23]. Studies have found that many prodromal patients ('at-risk' for psychosis) remain undetected in the community[24]; and there are few early identification programmes in operation.

Objectives

The key objectives of WIC are to:

a) develop a culturally tailored FEP management protocol, based on evidence synthesis of current clinical practice at partners sites and by modifying protocol from the NEDEN study, to make sustained health improvements for poorly served individuals with FEP.
b) better understand pathways of care to develop improved early detection strategies for psychosis relevant to India;
c) create a dataset of well-characterised inception cohorts of FEP with research-ready systems and processes, that will enable answer new research questions;
d) investigate clinical and cost-effectiveness of innovative community care models for psychosis including digital and mobile applications;
e) develop and implement school and college based intervention programs for early detection and referral of individuals with psychosis; and

For peer review only

f) build research capacity by providing a strong multidisciplinary research and training programme for researchers based in India.

A larger goal is to build strong research collaborations between India, UK and Canada partners for long-term research, training, knowledge exchange and service imporvement.

Methods

WIC Programme - Design

The design of WIC programme is depicted in Figure 1. Our strategic plan comprises five work packages (WP) and two cross-cutting themes (CTs) with clear objectives and deliverables. Additionally, we will exploit the potential of digital/mobile technologies to: (i) identify mental disorders in students, and (ii) increase access to clinical services and continuity of care for difficult-to-treat cases of schizophrenia. The study will be conducted in accordance with ethical principles that comply with the Declaration of Helsinki and the ICH Harmonised Tripartite Guideline for Good Clinical Practice. All participants will provide written informed consent or parental assent prior to entering a study.

WIC Programme - Management

The University of Warwick is the sponsor organisation. A programme manager (PM) is responsible for the day-to-day management of the research programme and supports the GHRG director for the overall delivery of the research programme. The PM, GHRG director and work package leads are responsible for the overall management of the study and meet monthly by video conference chaired by the director. The programme steering committee (PSC), comprising PM, GHRG director, co-investigators and WP leads provide overall strategic direction, ensure good governance, provide scientific input, guide capacity-building activities and provide financial probity when required. An independent external scientific advisory group (ESAG) comprising five international experts will monitor and assess the study conduct and progress. Both PSC and ESAG meet annually with the consortium to assess the scientific quality of research and to give feedback.

Study Sites

WIC brings together four institutions: University of Warwick, UK; McGill University, Canada; All India Institute of Medical Sciences (AIIMS), New Delhi, India; and Schizophrenia Research Foundation (SCARF), Chennai, India. SCARF, based in Chennai, is a non-profit voluntary organisation providing care to patients with

For peer review only

psychosis and a World Health Organisation (WHO)-collaborating centre for schizophrenia research. With a population of over 7 million, Chennai is the fourth largest metropolis in India. AIIMS, based in New Delhi with its metropolitan area population of 30 million, is a tertiary care hospital involved in providing undergraduate and postgraduate medical education and research, and hosts the National Drug Dependance Treatment Centre, a WHO collaborating centre.

Work Package 1:- Partnerships, collaborations and stakeholder engagement

In WP-1 will developed a network of external stakeholders in youth mental health including user and carer advocacy groups, clinicians, academicians and policy makers, and will be conducted at both study sites. A situational and needs analyses was conducted to identify user and carer priorities, barriers to help-seeking and modifiable aspects of care pathways. 'Therapeutic ingredients' of early intervention that can be introduced in clinical care in India will be identified. The results from this WP have been published elsewhere [25 26].

Work Package 2:- Protocol-based assessment and management of FEP

Utilising the insights from WP1 and evidence-based interventions developed in the UK and Canada for EIS, in WP2 we will adapt protocols from the UK NEDEN study [27], the largest FEP cohort in the world, to gather FEP cases at both SCARF and AIIMS, ensuring valid and reliable translation of assessment tools. A comprehensive package of biopsychosocial care, tailored to the local context will be developed and delivered to all new FEP cases and their short and medium-term outcomes measured. An FEP patient registry (comprising both affective and nonaffecrtive psychosis) will be maintained at our study sites. The study population, study assessment tools and study assessment schedules are described in Section A in the supplementary material.

Work Package 3:- Understanding pathways to care

The objective of WP3 is to determine pathways to care in FEP at both sites, using a combination of qualitative and quantitative approaches. We will explore DUP and its components (help-seeking delay, treatment delay following presentation) , cultural attribution of psychosis symptoms (e.g., individual, supernatural), help-seeking behaviour and modifiable factors that contribute to DUP. We will exploit the established methodology for case-ascertainment and care pathways from the NIHR ENRICH Programme [28]. We will examine the following questions:

For peer review only

I. Do the two cohorts (South India Vs North India) differ significantly in culturally mediated illness attributions (i.e., individual, natural, social, supernatural, and no attribution) during FEP? The cohorts vary demographically and in terms of study setting (outpatient NGO in South India vs. large general hospital with emergency, inpatient and outpatient facilities in North India).

II. Do the two cohorts differ in their pathways to care (i.e., DUP, compulsory detention, psychiatrists, emergency services, general practitioner, faith-based organisations) during FEP?

III. Are the pathways to care during FEP influenced by culturally mediated illness attributions and other relevant socio-demographic (i.e., gender, religious practice, marital status, living alone, education, income) and clinical confounders such as DUP?

Study setting, population and study assessment tools: This study will be conducted at SCARF, Chennai and AIIMS, New Delhi, using the datasets collected from FEP patients recruited as part of WP2. The study instruments that will be used in this study are: (i) *The Emerging Psychosis Attribution Schedule* (EPAS) [29]; (ii) Nottingham Onset Schedule (NOS) [30]; and (iii) an amended version of the *Encounter form* by Gater and Sousa [29].

Work Package 4:- Mobile technology to ensure continuity of care

Globally, India has the second largest number of mobile phone users – 900 million users accounting for 88% of all telecommunication users [31]. Studies have emerged from high income countries on mobile technologies in psychosis [32] but are lacking in LMICs [33]. Our preliminary survey shows that 88% of patients have mobile phones [34]. The objective of WP4 is to develop a home-based psycho-social care model for 'difficult-to-treat' patients with schizophrenia aimed at functioning and recovery, and an associated mobile-based application. In the absence of state provision in India, mobile-based clinical care has the potential to facilitate appropriate clinical care. In this WP,we will compare and evaluate the feasibility and effectiveness of a paper-version of the model with the mobile app in improving patient outcomes and reducing carer burden. This study will be conducted at AIIMS, New Delhi. The study population, inclusion/exclusion criteria, methodology, assessment tools and study assessment schedules are described in Section B of the supplementary material.

For peer review only

Work Package 5:-Early detection using a community awareness programme

The primary aim of WP5 is to develop and implement a school and college-based intervention for early detection and referral of individuals with emerging mental health problems. We wish to pilot a school- and college-based programme of online screening for emerging mental health problems; assess the effectiveness of using community resources (teachers, peers and youth groups) to provide psycho-social intervention; and determine the clinical and cost effectiveness of the screening programme. This study will be conducted by SCARF in Chennai, India. The identified catchment area for the study is Anna Nagar, Chennai and adjacent areas that have fourteen schools and four colleges. Informed consent from the management of the educational institutions will be sought before conducting the study at their institution. The study assessment tools and study intervention are described in Section C of the supplementary material.

Two Cross Cutting Themes (CT)

Implementation Science

Despite being the first LMIC to introduce a National Mental Health Programme, there remains a significant implementation gap in the delivery of evidence-based interventions for mental health care in India [35]. [36]. Implementation science represents an approach focused upon understanding the challenges, identifying the barriers and harnessing facilitators to help translate research-based evidence to 'real-world' clinic and community settings, developing leadership and changing professional practice to support delivery of evidence-based innovation [37].

The primary objective of this study is toevaluate the implementation of the early intervention in psychosis programme (EIS) in India. We will address the implementation barriers and facilitators in healthcare according to the Consolidated Framework for Implementation Research (CFIR) [38]. Our specific aims are: (i) to identify barriers and facilitators to implementation, sustaining (beyond pilot) and scaling up of evidence-based early intervention to address psychosis; (ii) to assess how the original intervention is adapted to fit context, and to what extent it then diverges from its original evidence base. The study will be carried out at SCARF, Chennai.

Health Economics

The primary objective of the health economics theme is to assess the cost of treatment for patients with FEP attending AIIMS from the payer and provider perspective. We will develop and pilot a questionnaire to determine direct and indirect costs

For peer review only

experienced by patients with mental illness, and their households, and the coping strategies used in response to these costs. From these data, we will estimate mean, median and quartile costs at each time point. We will also conduct a bottom-up costing of the typical package of care offered to those participating in WP2 at AIIMS. This will be based on unit cost data collected during the study, along with interviews with clinicians taking part in the study on the care delivered, and the time taken to deliver it.

Capacity Building Activities

There is an urgent need to develop and deliver high quality research capacity-building activities in India, ranging from modules on research methodology to doctoral level training and courses aimed at healthcare and leadership.

PhDs

WIC, along with TATA Trust, India and Warwick Medical School are supporting three PhD candidates from India at the University of Warwick, whilst enabling them to carry out their primary research activities in their home countries. These three students, who were selected after a competitive process, commenced their doctoral studies in May 2019. The PhD topics included projects that are nested within WIC and stand-alone projects that are closely aligned to the WIC research priorities. At least two University of Warwick-based supervisors and one partner country-based supervisors are allocated to each student for their PhDs. Topics approved for the first cohort of WIC PhD students include: (i) health economics; (ii) pathways of care and service engagements in early intervention services; and (iii) assessing factors associated with hospital re-admissions and strategies to reduce the burden of re-admissions in psychiatric hospitals in India.

Research leadership training programme

At Warwick Business School (WBS), we already have an international distance learning *Leadership Development Programme (LDP)* for clinicians, with case studies from Africa and South East Asia. We will develop a contextualized, distance-learning intervention about leadership and innovation for mental health professionals in India. This innovative training will be focussed on developing another case from the WIC programme, specific to youth mental health in India. Integrating leadership and innovation training in clinical and service development domains will be beneficial for mental health professionals, particularly in formulating clinical priorities and engaging meaningfully with policy makers in the country.

For peer review only

Planned Extensions

The WIC GHRG has secured funding from NIHR to conduct an additional programme of work, which builds on the existing programme of work, for a duration of up to 12 months. In this costed extension programme, we will: (i) extend our current school-based screening for mental disorders (WP5) in two vulnerable populations: a) refugee youth (Jammu & Kashmir); and b) a high-risk tribal population in Mahrashtra; (ii) expand our FEP registry (WP2) to a rural setting (Nagpur), for potential comparisons with other sites and with existing datasets and including additional research questions of interest (such as cardiometabolic parameters, life style interventions); (iii) extend health economic analysis to include rural populations and explore how financial burden and coping mechanisms differ between settings; and (iv) develop a research-based leadership training programme.

Analysis plan

For WP-1, a prospective cross-sectional study in the form of a situational analysis and needs assessment with mental health professionals and external stakeholders will be conducted to capture information on current practices and available resources for the management of FEP.A semi-structred pro-forma will be developed to capture quantitative and qualtitative data on facilities available for FEP management at each health centres, pharmacological and non-pharmacological management, local training needs, hospitalization data and follow-up data of FEP cases. The data will be analysed using standard summary statistsical methods, with indicators of variability, central tendancy, frequency, percentage, ratio or proportion, as appropriate

For the remaining WP's and CTs, we will use standard descriptive statistical methods to evaluate the data. Where appropriate, continuous variables will be assessed for normality, and non-normally distributed variables will be transformed or assessed using non-parametric tests as appropriate. To compare the data (e.g. sociodemographic and clinical factors) between and within study sites, we will use independent sample *t*-tests for continuous variables and χ^2 for dichotomous variables (WP-2 & WP-3). To assess the change in several domains of symptom severity and functioning at pre-specified time points, we will use a general linear modelling analysis, adjusting for various potential confounders (e.g. WP-2, WP-4). We will use test adjusted and unadjusted associations using appropriate statistical tests as appropriate (e.g. WP-3). For unadjusted analysis: (i) ANOVA for testing unadjusted associations between ethinic groups and culturally mediated illness attributions and service

encounters (normally distributed continuous outcomes); (ii) Kruskal – Wallis rank sum tests (for non-normally distributed continuous outcomes); and (iii) logistic regression for dichotomous variables. For adjusted association analysis, we will use multiple logistic regression analysis, controlling for various confounders of interest. To provide information regarding the magnitude of the change in scores in domains of interest (e.g. WP-5), *Cohen's d* effect sizes will be calculated with effect size values of ≥0.5, ≥0.8 and ≥1.0 corresponding to a moderate, large, and very large effect, respectively n(e.g WP-5). For CTs such as implementation science and health economics, we will use standard descriptive statistics to describe the data and framework analysis (implementation science), if deemed necessary.

Statistical significance will be inferred at $P < 0.05$. We will consider sensitivity to change as adequate, if the improvement is statistically significant ($P < 0.05$) and with at least a moderate effect size (Cohen's d ≥ 0.50). The primary analysis will be undertaken by excluding patients with missing data. However, we will conduct sensitivity analysis for missing data using the following imputation techniques, as appropriate: (i) last value carried forward (LVCF); (ii) baseline value carried forward (BVCF); (iii) single imputation with EM algorithm; and (iv) multiple imputation. Data will be analysed using appropriate statistical software (eg SPSS, R, STATA).

Assuring instrument reliability

Clinical instruments requiring ratings by research staff will continue to be tested for inter-rater reliability across the two sites, SCARF and AIIMS. A joint meeting of the research staff from both sites was held and inter-rater reliability exercises on the important measures were conducted using video-recorded interviews with 10 randomly selected patients with FEP. Data was successfully collected for the following measures: Scale for Assessment of Positive Symptoms, Scale for The Assessment of Negative Symptoms, Young Mania Rating Scale, Montgomery-Asberg Depression Rating Scale, Brief Psychiatric Rating Scale, Global Assessment of Functioning, Social and Occupational Functioning Assessment Scale and Medication Adherence Rating Scale. A high degree of inter-rater reliability across sites (intraclass correlations from 0.74 to 0.92) was obtained on these measures. Our results were comparable with previous literature [39,40].

For peer review only

Ethics and dissemination

Ethics

Written, informed consent will be obtained from all participants or carers as appropriate, before their enrolment into the study. In the school and college-based study (WP-5), all participants will receive information on where to seek help for mental health problems. In addition, we have developed a clinical care pathway for those who are identified as being at ‘high-risk for severe mental illness or having undetected /untreated severe mental illness or with suicidal ideations that will ensure safety, protection of confidentiality and privacy. The study principal investigator and team will preserve the confidentiality and anonymity of participants taking part in the study and fulfil transparency requirements under the General Data Protection Regulation for health and care research. The study may be subject to inspection and audit by the University of Warwick under their remit as sponsor and other regulatory bodies to make sure adherence to GCP and the UK Policy Framework for Health and Social Care Research.

This study has been approved by the University of Warwick’s Biomedical and Scientific Research Ethics Committee (reference: REGO-2018-2208), Coventry, UK and research ethics committees of all participating organisations. The ethical conduct of the study is monitored throughout by the University of Warwick and each participating organisation.

Dissemination Strategies

We will strategically exploit our strong network with various LMIC stakeholders involved in mental health services such as clinicians, academics, service providers, policy makers, government and non-governmental agencies, community groups, patient and carer advocacy groups, media agencies and the creative arts industry to support WIC dissemination activities. We will use a variety of methods to ensure effective dissemination of the groups’ work including; (a): development of an up-to-date project website (to target different audiences/stakeholders) (b) presentations at national and international psychiatric and mental health scientific conferences; peer review publications, and (c) publication through visual media including film and theatre. Dissemination activities through cinema and theatre have a particular resonance for many LMIC populations and we believe that we will reach a large number of people through these activities. We will work closely with our LMIC partners to identify the dissemination approaches that might work best in this setting.

For peer review only

Patient and Public Involvement

We have strong and ongoing engagement with various internal and external stakeholders involved in mental healthcare.The WP-1 of WIC uses focus groups as a method for situational analysis and needs analysis and will include patients and carer groups, and voluntary/charity/third-sector organsiations, ensuring representation of hard-to-reach groups. Patient and/or public groups were not involved in proposing the research objectives nor involved in the overall study design or the implementation of this research programme. However, stakeholders involved in mental health will be engaged by the research team at respective sites as local dissemination plans are being devised. All partners also have an extensive record of patient and public involvement (PPI) in research. We have a robust programme for monitoring and governance-research, ethics, financial and our programme will adhere to the highest levels of ethical practice and probity including PPI involvement.

Conclusion

Although major improvements have been achieved in the treatment of patients with psychotic disorders in high-income countries including the widescale implementation of EIS in psychosis, implementing such services remains a major challenge in LMICs. The sizeable differences in patient profiles, socio-demographics, pathways of care, barriers to care,comorbidities, scarcity of resources and delayed presentation means, at the very least, Western models of care cannot be simply translocated to LMICs. The WIC programme will tailor evidence-informed interventions to the Indian socio-cultural context to address the increasing burden of psychotic disorders in India. By using a bespoke interventional approach that is regionally relevant, and by exploiting the untapped potential of digital and mobile technologies in India, WIC will develop and test interventions that can be integrated into existing clinical care, and generate data (including health economic data) that will inform priorities for healthcare providers and policy makers.

For peer review only

Disclaimer: This research was commissioned by the National Institute of Health Research using Official Development Assistance (ODA) funding. The views expressed are those of the author(s) and not necessarily those of the NIHR or the Department of Health and Social Care.

[illegible]

Funding: United Kingdom's National Institute of Health Research (NIHR) using Official Development Assistance (ODA) funding (Award number: 16/137/107).

[illegible]

Author Contributions: SPS is the Director of this global health research group. SPS conceived the original study design and obtained funding. MM is the programme manager and prepared the first draft and subsequent revisions of the manuscript, and is the joint first author with SPS. TR,PR, MS, JM, GC and RKC contributed writing to particular sections relevant to their work package of the Group. SI,CM,JS,MB, RL and VF provided critical comments during the proposal-writing stage, WIC project meetings and critically reviewed and revised the manuscript. All authors read and approved the final manuscript.

Conflicts of Interests

None

Data Statement

Data will be stored in University of Warwick repository for at least 10 years after its last access, in accordance with Research Data Management Policy, and will be made available to other users under data-sharing agreement. The decision on whether to share research samples and/or supply data to a potential new user will be made by the Programme Management Group (PMG). All data requests should be submitted to the chief investigator for consideration.

Acknowledgements

We thank the WIC independent external scientific advisory group (ESAG), for providing strategic insight and international perspectives for WIC and also for their valuable inputs to the critical decisions affecting the scientific orientation of the WIC research programme. S.P.S and M.B are supported by the NIHR Applied Research Centre (ARC) West Midlands. S.N.I is supported by a salary award from the Canadian Institutes of Health Research. J.S. is supported by a clinician-scientist salary award from the Fonds de Recherche du Québec – Santé.

For peer review only

Ethics Statements

Patient consent for publication

Not required

Ethics approval

This study has been approved by the University of Warwick's Biomedical and Scientific Research Ethics Committee (reference: REGO-2018-2208), Coventry, UK; SCARF, Chennai, India; and AIIMS, New Delhi India.

For peer review only

References

1. Collaborators GDaIIaP. Global, regional, and national incidence, prevalence, and years lived with disability for 354 diseases and injuries for 195 countries and territories, 1990-2017: a systematic analysis for the Global Burden of Disease Study 2017. Lancet 2018;392(10159):1789-858. doi: 10.1016/S0140-6736(18)32279-7 [published Online First: 2018/11/08]

2. Farooq S. Early intervention for psychosis in low- and middle-income countries needs a public health approach. Br J Psychiatry 2013;202(3):168-9. doi: 10.1192/bjp.bp.112.113761

3. Rathod S, Pinninti N, Irfan M, et al. Mental Health Service Provision in Low- and Middle-Income Countries. Health Serv Insights 2017;10:1178632917694350. doi: 10.1177/1178632917694350 [published Online First: 2017/03/28]

4. Shiers D, Lester H. Early intervention for first episode psychosis. BMJ 2004;328(7454):1451-2. doi: 10.1136/bmj.328.7454.1451 [published Online First: 2004/06/19]

5. Sudharsanan N BD. The Demography of Aging in Low- and Middle-Income Countries: Chronological versus Functional Perspectives. . M Majumdar, M Hayward, M Majumdar, M Hayward (Eds), Future directions for the demography of aging (1st ed), National Academies Press, Washington, DC 2018:pp. 309-38.

6. Chatterjee S, Pillai A, Jain S, et al. Outcomes of people with psychotic disorders in a community-based rehabilitation programme in rural India. Br J Psychiatry 2009;195(5):433-9. doi: 10.1192/bjp.bp.108.057596

7. Malla A, McGorry P. Early Intervention in Psychosis in Young People: A Population and Public Health Perspective. Am J Public Health 2019;109(S3):S181-S84. doi: 10.2105/AJPH.2019.305018 [published Online First: 2019/06/27]

8. Correll CU, Galling B, Pawar A, et al. Comparison of Early Intervention Services vs Treatment as Usual for Early-Phase Psychosis: A Systematic Review, Meta-analysis, and Meta-regression. JAMA Psychiatry 2018;75(6):555-65. doi: 10.1001/jamapsychiatry.2018.0623 [published Online First: 2018/05/26]

9. Chiliza B, Asmal L, Emsley R. Early intervention in schizophrenia in developing countries: focus on duration of untreated psychosis and remission as a treatment goal. Int Rev Psychiatry 2012;24(5):483-8. doi: 10.3109/09540261.2012.704873

For peer review only - http://bmjopen.bmj.com/site/about/guidelines.xhtml

10. McGorry PD, Ratheesh A, O'Donoghue B. Early Intervention-An Implementation Challenge for 21st Century Mental Health Care. JAMA Psychiatry 2018;75(6):545-46. doi: 10.1001/jamapsychiatry.2018.0621

11. Singh SP. Early intervention in psychosis. Br J Psychiatry 2010;196(5):343-5. doi: 10.1192/bjp.bp.109.075804

12. McGorry PD, Hartmann JA, Spooner R, et al. Beyond the "at risk mental state" concept: transitioning to transdiagnostic psychiatry. World Psychiatry 2018;17(2):133-42. doi: 10.1002/wps.20514

13. Chokshi M, Patil B, Khanna R, et al. Health systems in India. J Perinatol 2016;36(s3):S9-S12. doi: 10.1038/jp.2016.184

14. Lamers LM, McDonnell J, Stalmeier PF, et al. The Dutch tariff: results and arguments for an effective design for national EQ-5D valuation studies. Health Econ 2006;15(10):1121-32. doi: 10.1002/hec.1124

15. Singh SP. Early intervention in the Indian context: In S. Malhotra & S. Chakrabarti (Eds.) 2015:89-98.

16. Large M, Farooq S, Nielssen O, et al. Relationship between gross domestic product and duration of untreated psychosis in low- and middle-income countries. Br J Psychiatry 2008;193(4):272-8. doi: 10.1192/bjp.bp.107.041863

17. Chadda RK, Agarwal V, Singh MC, et al. Help seeking behaviour of psychiatric patients before seeking care at a mental hospital. Int J Soc Psychiatry 2001;47(4):71-8. doi: 10.1177/002076400104700406

18. Hanna F, Barbui C, Dua T, et al. Global mental health: how are we doing? World Psychiatry 2018;17(3):367-68. doi: 10.1002/wps.20572

19. Garg K, Kumar CN, Chandra PS. Number of psychiatrists in India: Baby steps forward, but a long way to go. Indian J Psychiatry 2019;61(1):104-05. doi: 10.4103/psychiatry.IndianJPsychiatry_7_18

20. Gururaj G VM, Benegal V, Rao GN, Pathak K, Singh LK, Mehta RY, Ram D,, Shibukumar TM KA, Lenin Singh RK, Chavan BS, Sharma P, Ramasubramanian, C DP, Saha PK, Deuri SP, Giri AK, Kavishvar AB, Sinha VK, Thavody J, Chatterji R,, et al. National Mental Health Survey of India, 2015-16: Summary. Bengaluru, National

Institute of Mental Health and Neuro Sciences. Bengaluru: NIMHANS, 2016.

For peer review only - http://bmjopen.bmj.com/site/about/guidelines.xhtml

21. Saxena S, Sharan P. [Supporting mental health research publications from low- and middle-income countries]. Braz J Psychiatry 2004;26(2):73-4. doi: 10.1590/s1516-44462004000200001 [published Online First: 2004/11/02]

22. Chadda RK. Caring for the family caregivers of persons with mental illness. Indian J Psychiatry 2014;56(3):221-7. doi: 10.4103/0019-5545.140616 [published Online First: 2014/10/16]

23. Koschorke M, Padmavati R, Kumar S, et al. Experiences of stigma and discrimination of people with schizophrenia in India. Soc Sci Med 2014;123:149-59. doi: 10.1016/j.socscimed.2014.10.035 [published Online First: 2014/10/18]

24. Keshavan MS, Shrivastava A, Gangadhar BN. Early intervention in psychotic disorders: Challenges and relevance in the Indian context. Indian J Psychiatry 2010;52(Suppl 1):S153-8. doi: 10.4103/0019-5545.69228

25. Dhandapani VR, Ramachandran P, Mohan G, et al. Situational analysis of prevailing practices in the management of first-episode psychosis in Chennai, India. Early Interv Psychiatry 2020 doi: 10.1111/eip.12979 [published Online First: 2020/05/21]

26. Dhandapani VR, Chandrasekaran S, Singh S, et al. Community stakeholders' perspectives on youth mental health in India: Problems, challenges and recommendations. Early Interv Psychiatry 2020 doi: 10.1111/eip.12984 [published Online First: 2020/05/21]

27. Birchwood M, Lester H, McCarthy L, et al. The UK national evaluation of the development and impact of Early Intervention Services (the National EDEN studies): study rationale, design and baseline characteristics. Early Interv Psychiatry 2014;8(1):59-67. doi: 10.1111/eip.12007 [published Online First: 2013/01/26]

28. Singh SP, Islam Z, Brown LJ, et al. Ethnicity, detention and early intervention: reducing inequalities and improving outcomes for black and minority ethnic patients: the ENRICH programme, a mixed-methods study2013.

29. Singh SP, Brown L, Winsper C, et al. Ethnicity and pathways to care during first episode psychosis: the role of cultural illness attributions. BMC Psychiatry 2015;15:287. doi: 10.1186/s12888-015-0665-9 [published Online First: 2015/11/16]

For peer review only - http://bmjopen.bmj.com/site/about/guidelines.xhtml

30. Singh SP, Cooper JE, Fisher HL, et al. Determining the chronology and components of psychosis onset: The Nottingham Onset Schedule (NOS). Schizophr Res 2005;80(1):117-30. doi: 10.1016/j.schres.2005.04.018 [published Online First: 2005/06/22]

31. Stalin P, Abraham SB, Kanimozhy K, et al. Mobile Phone Usage and its Health Effects Among Adults in a Semi-Urban Area of Southern India. J Clin Diagn Res 2016;10(1):LC14-6. doi: 10.7860/JCDR/2016/16576.7074 [published Online First: 2016/01/01]

32. Firth J, Torous J. Smartphone Apps for Schizophrenia: A Systematic Review. JMIR Mhealth Uhealth 2015;3(4):e102. doi: 10.2196/mhealth.4930 [published Online First: 2015/11/06]

33. Sood M, Chadda RK, Singh P. Mobile health (mHealth) in mental health: Scope and applications in low-resource settings. Natl Med J India 2016;29(6):341-43.

34. M Sood RC, K Sinha Deb, R Bhad , A Mahapatra, R Verma, AK Mishra. Scope of Mobile Phones in Mental Health Care in Low Resource Settings. Journal of Mobile Technology in Medicine 2016(5:2:33–37)

35. van Ginneken N, Jain S, Patel V, et al. The development of mental health services within primary care in India: learning from oral history. Int J Ment Health Syst 2014;8:30. doi: 10.1186/1752-4458-8-30 [published Online First: 2014/07/16]

36. Shidhaye R. Implementation Science for closing the treatment gap for mental disorders by translating evidence base into practice: experiences from the PRIME project. Australas Psychiatry 2015;23(6 Suppl):35-7. doi: 10.1177/1039856215609771

37. Yapa HM, Bärnighausen T. Implementation science in resource-poor countries and communities. Implement Sci 2018;13(1):154. doi: 10.1186/s13012-018-0847-1 [published Online First: 2018/12/27]

38. Damschroder LJ, Aron DC, Keith RE, et al. Fostering implementation of health services research findings into practice: a consolidated framework for advancing implementation science. Implement Sci 2009;4:50. doi: 10.1186/1748-5908-4-50 [published Online First: 2009/08/07]

39. Iyer SN, Mangala R, Thara R, et al. Preliminary findings from a study of first-episode psychosis in Montreal, Canada and Chennai, India: comparison of outcomes. Schizophr Res 2010;121(1-3):227-33. doi: 10.1016/j.schres.2010.05.032

For peer review only - http://bmjopen.bmj.com/site/about/guidelines.xhtml

40. Malla A, Iyer SN, Rangaswamy T, et al. Comparison of clinical outcomes following 2 years of treatment of first-episode psychosis in urban early intervention services in Canada and India. Br J Psychiatry 2020:1-7. doi: 10.1192/bjp.2020.126 [published Online First: 2020/07/06]

For peer review only

For peer review only - http://bmjopen.bmj.com/site/about/guidelines.xhtml

Figure Legends:

Figure 1: Schematic Overview of WIC GHRG. WP, Work Package

For peer review only

For peer review only - http://bmjopen.bmj.com/site/about/guidelines.xhtml

For peer review only

For peer review only - http://bmjopen.bmj.com/site/about/guidelines.xhtml

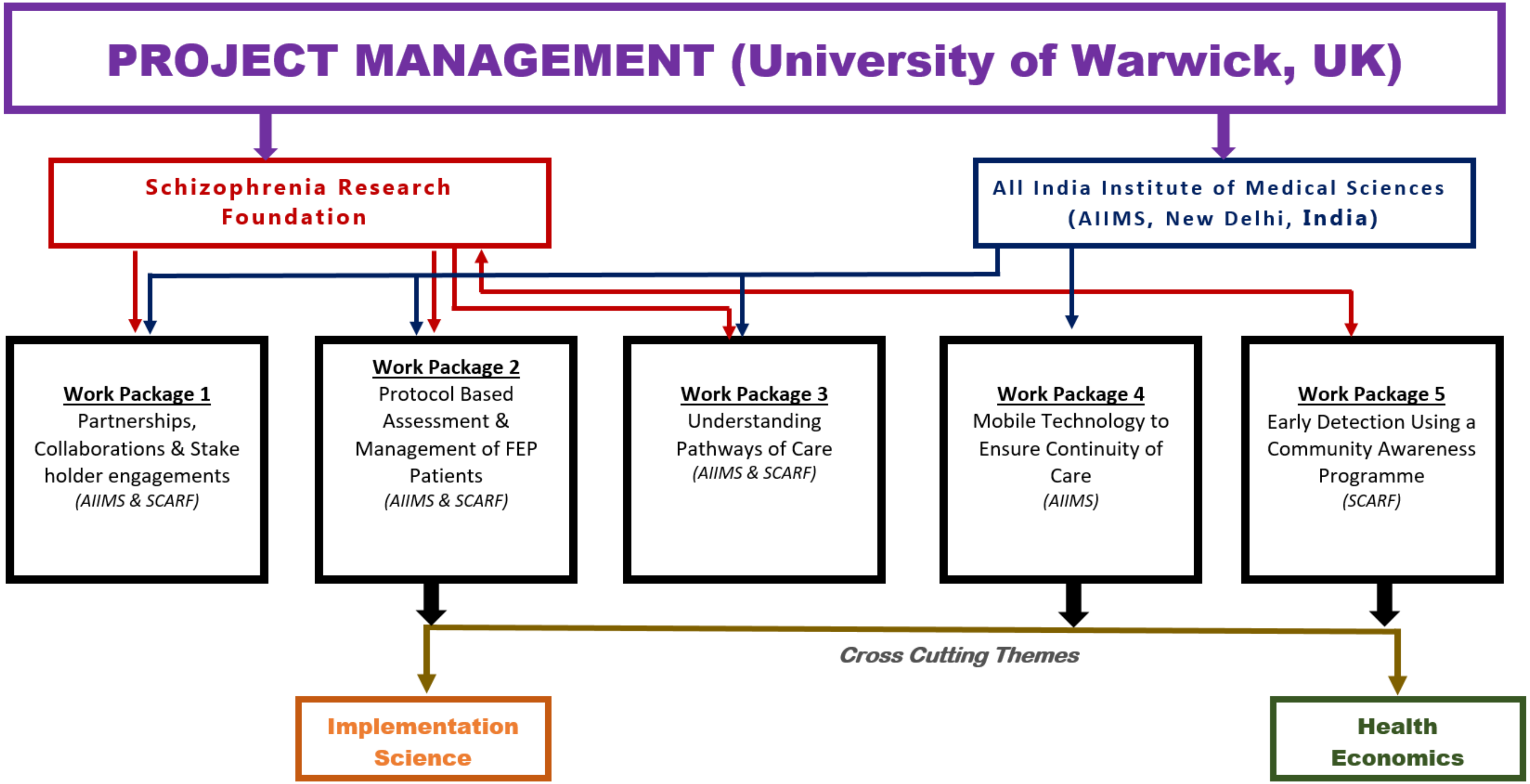

Figure 1: Schematic Overview of WIC GHRG. WP, Work Package

For peer review only

For peer review only - http://bmjopen.bmj.com/site/about/guidelines.xhtml

Supplementary Material – WIC

For peer review only

For peer review only - http://bmjopen.bmj.com/site/about/guidelines.xhtml

Section A – *Work Package 2:- Protocol-based assessment and management of FEP*

Study Population

Patients with 'untreated' FEP and their carers visiting psychiatry department at respective collaborating sites, fulfilling the study inclusion/exclusion criteria will be recruited into the study. Participating patients must be aged 16–45 years with a diagnosis of FEP based on the International Classification of Diseases, 10th revision (ICD-10) codes for schizophrenia (F20), persistent delusional disorder (F22), acute and transient psychotic disorders (F 23), schizoaffective disorder (F25), other and unspecified psychosis (F28-29), mania with psychotic symptoms (F30.2), bipolar affective disorder, current episode manic with psychotic symptoms (F 31.2), severe depressive episode with psychotic symptoms (F32.3) and recurrent depressive disorder and/or current episode depression with psychotic symptoms (F 33.3). Untreated is defined as not having received anti-psychotic medication for more than 30 days since the onset of psychosis. Patients must be able to communicate in Tamil or English in Chennai, and Hindi or English in New Delhi. Study exclusion criteria are: those with overt learning disability (equivalent to IQ <70, ascertained either by previous diagnosis or *detailed history from the patients and their caregivers, corroborated by observations from academic performance assessed using test scores from various subjects (such as language and mathematics)*, organic brain damage and epilepsy of pervasive developmental disorder.

Written informed consent will be obtained from interested and eligible participants prior to participation. Consent will also be sought from interested carers of study participants for carer-reported measures.

Study Assessment Tools

Various clinical scales will be used to assess several domains of symptom severity and patient functioning at baseline and follow-up assessments. Baseline assessment will be conducted soon after entry into the service, and follow-up assessments will be conducted at 3, 6 and 12 months.

Socio-demographic, clinical, and socio/family variables will be collected using semi-structured (MRC demographic schedule) face-to-face clinical interview. Psychopathology will be assessed using the: (i) Scale for the Assessment of Positive Symptoms (SAPS) (1); (ii) Scale for the Assessment of Negative Symptoms (SANS) (1); (iii) Young Mania Rating Scale (YMRS) (2); (iv) Montgomery - Asberg Depression

For peer review only - http://bmjopen.bmj.com/site/about/guidelines.xhtml

Rating Scale (3); and (v) Brief Psychiatric Rating Scale (BPRS) (4). Severity of illness and functioning will be measured using the Global Assessment of Functioning (GAF) Scale (5), the Social And Occupational Functioning Assessment Scale (SOFAS) (6) and 5-level EQ-5D (EQ 5 D 5L) (7). Other assessment tools that will be used in this study are: (i) The Emerging Psychosis Attribution Schedule (EPAS) to assess patient and carer attributions of symptoms in the emerging phase of a psychotic episode (8); (ii) the Nottingham Onset Schedule (NOS) to establish duration of untreated psychosis (9); (iii) an amended version of the Encounter form to assess service encounters during the psychotic phase of the illness (8); (iv) Medication Adherence Rating Scale (MARS) for assessment of medication adherence in psychosis(10); (v) an assessment schedule to assess the nature of insight in psychosis (11); (vi) tools for assessment of risk factors (trauma exposure) including the Childhood Trauma Questionnaire (CTQ) (12), Harvard Trauma Questionnaire (HTQ) (13) and the List Of Threatening Experiences (LTE) (14); and (vii) Brief Assessment of Cognition in Schizophrenia (BACS) for neurocognition. Additionally, we will also capture data on treatment, relapse and physical health. The caregivers will be assessed on the Burden Assessment Schedule (BAS) (15). Engagement of caregivers will be encouraged to obtain accurate patient-information including patients' medical history, risk factors, family responses to illness, help-seeking and potential impact on family. *The estimated time to complete the study assessments will be around 60 min - 90 min. Each assessment will be conducted in two or more sessions depending on the participant's time, availability and comfort.* Supplementary table 1 depicts the assessment schedule for both baseline and follow-up assessments.

Intervention and Management of FEP

Management of FEP

Following a comprehensive assessment as described above, patients with FEP will be offered the following treatment options:

Antipsychotics

- Oral antipsychotics in lowest possible dose taking into account of effectiveness, tolerability and patient preference.
- Depot antipsychotics may be used if adherence is a significant problem or based on patient preference.

For peer review only

- The choice of antipsychotic medication will be discussed by the service user and healthcare professional, and decision will be made, taking into account of the views of the carer. The study team will provide more information on antipsychotics of choice and discuss the likely benefits and possible side effects of each drug, including:
    - metabolic (including weight gain and diabetes)
    - extrapyramidal (including akathisia, dyskinesia and dystonia)
    - cardiovascular (including prolonging the QT interval)
    - hormonal (including increasing plasma prolactin)
    - other (including unpleasant subjective experiences)

Before starting antipsychotic medication, the study team will undertake and record the following baseline investigations:

- weight and BMI
- waist circumference
- pulse and blood pressure
- fasting blood glucose, glycosylated haemoglobin (HbA_{1c}), blood lipid profile and prolactin levels
- assessment of any movement disorders
- assessment of nutritional status, diet and level of physical activity.
- Before starting antipsychotic medication, offer the person with psychosis or schizophrenia an electrocardiogram (ECG) if:
- specified in the summary of product characteristics (SPC)
- a physical examination has identified specific cardiovascular risk (such as diagnosis of high blood pressure)
- there is a personal history of cardiovascular disease

Psychosocial interventions

For Patients

- Psycho-education
- Counselling
- Supportive psycho therapy
- Cognitive behavioral therapy

For peer review only - http://bmjopen.bmj.com/site/about/guidelines.xhtml
For peer review only

- Relapse prevention therapy
- Group therapy
- Vocational/occupational therapy
- Net working

For Family

- Psycho education
- Family intervention
- Family counselling
- Family therapy

Monitoring and follow-up

- Patients will be assessed formally 3, 6 and 12 months according to the schedule in Fig 1.
- Response to treatment, including symptom and behavioural change, adherence to treatment, relapse, crises and hospitalisations will be recorded at every follow-up
- Weight, BMI, blood pressure, blood glucose and lipids will be monitored at 3, 6 and 12 months and preventive strategies implemented (life style and dietary change, metformin etc.)

For peer review only - http://bmjopen.bmj.com/site/about/guidelines.xhtml

Supplementary Table 1: Study Assessment Schedule of WIC

Study Instruments	Baseline	Month 3	Month 6	Month 12
MRC Sociodemographic Schedule	X	-	-	-
Nottingham Onset Schedule (NOS)	X	-	-	-
Emerging Psychosis Attribution Schedule (EPAS)	X	-	-	-
Service Encounters Interview	X	-	-	-
Scale for Assessment of Positive Symptoms (SAPS)	X	X	X	X
Scale for Assessment of Negative Symptoms (SANS)	X	X	X	X
Young Mania Rating Scale (YMRS)	X	X	X	X
Montgomery-Asberg Depression Rating Scale	X	X	X	X
Brief Assessment of Cognition in Schizophrenia (BACS)	X	X	X	X
Global Assessment of Functioning(GAF)	X	X	X	X
Social and Occupational Functioning Assessment Scale (SOFAS)	X	X	X	X
Brief Psychiatric Rating Scale (BPRS)	X	X	X	X
Medication Adherence Rating Scale (MARS)	X	X	X	X
EQ-5D 5 L	X	X	X	X
Burden Assessment Schedule (BAS) – Carer	X	X	X	X
Risk Factor Assessment (CTQ, HTQ & LTE)	X	-	-	-
Physical Health Assessment, treatment, relapse*	X	X	X	X
Analysis of Cost*	X	X	X	X

**Investigator designed ; CTQ, childhood trauma questionnaire; HTQ, Harvard trauma questionnaire; LTE, list of threatening experiences (LTE)*

For peer review only - http://bmjopen.bmj.com/site/about/guidelines.xhtml

Section B – Work Package 4:- Mobile technology to ensure continuity of care (Commissioned Project)

Background

In India, over 90% of patients stay in the community with families, and in the absence of state provision, families initiate treatment, procure and supervise medicines and provide psychosocial support including opportunities for education, vocation and recreation (16). Importantly, the stress of caregiving adversely affects carer's' physical and mental health(17). In this WP, we will deliver a home-based psycho-social recovery care model, strategies for reducing carer burden, and evaluate its clinical and cost-effectiveness using a three-pronged approach (Supplementary figure 2). We will deliver a mobile application for home-based psychosocial recovery, strategies for reducing carer burden, and determine its clinical and cost-effectiveness.

In brief, first, we will conduct focus group discussions with patients with schizophrenia, their families/caregivers and mental health professionals, to gather information on their healthcare needs and expected outcomes from a home-based psycho-social care model. Second, utilizing insights from these focus groups and an extensive literature review, we will develop a 'bespoke' home-based psycho-social care model (named as SAKSHAM, meaning effort in Hindi, booklet version) aimed at improving functioning and recovery of 'difficult-to-treat' patients with schizophrenia; and at reducing carer burden.

The Saksham will have two parts: booklets and mobile app. These will be developed after conducting focussed group discussions (FGDs) with stakeholders: patients, family caregivers and mental health professionals. Based on existing evidence and themes developed from the FGDs, the domains identified for the psychosocial intervention were medication adherence, physical health monitoring, activities of daily living, instrumental activities of daily living, diet management and need for psychoeducation. Intervention package for all six target domains will be developed. The content of the Saksham booklets will be in Hindi and English for both patients and caregivers. The content validity of the booklets will be tested by getting inputs from the stakeholders. The content of the booklets will be digitised for the Saksham mobile application. Finally, we will evaluate the feasibility and effectiveness of both the booklet version and mobile based application in a small group of patients with schizophrenia and their caregivers, and will compare against treatment as usual

For peer review only - http://bmjopen.bmj.com/site/about/guidelines.xhtml

group. This study will be conducted at AIIMS, New Delhi, India. For an overview of study inclusion/exclusion criteria of study cohort and list of study instruments with assessment schedule, see Supplementary Table 2 and Supplementary Table 3, respectively.

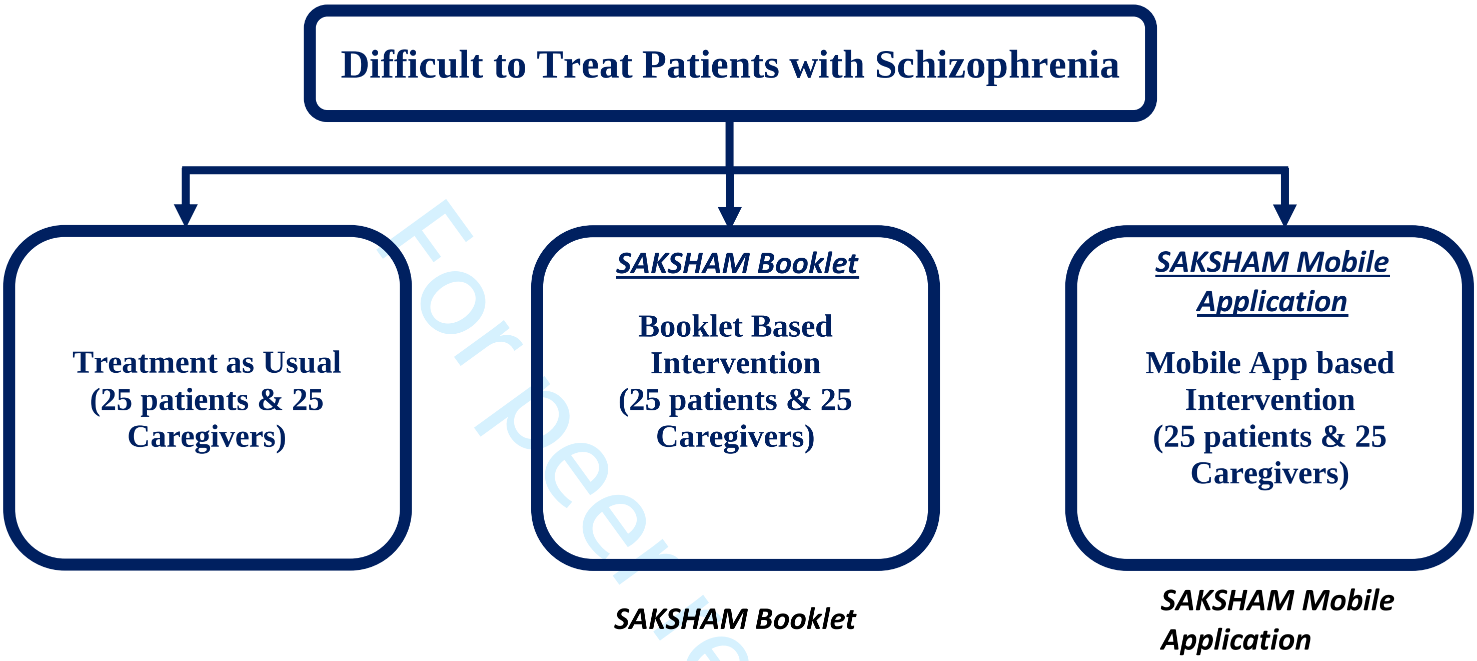

Supplementary Figure 1: Three arm study to evaluate the feasibility and effectiveness of home-based psycho-social recovery model.

For peer review only - http://bmjopen.bmj.com/site/about/guidelines.xhtml

Supplementary Table 2 : Study Inclusion /Exclusion Criteria for Patients and their Caregivers.

Patients	Caregivers
Inclusion Criteria	***Inclusion Criteria***
Aged 18–65 years	Immediate family member fulfilling any three of following criteria:<br>• Spending maximum time with the patient.<br>• Supporting the patient financially<br>• Most involved in the care of the patient<br>• Able to be contacted by treatment staff in case of an emergency
Diagnosis of schizophrenia based on International Classification of Diseases - Tenth Revision (ICD-10)codes [Schizophrenia (F20), Persistent delusional disorder (F22), Schizoaffective disorder (F25), Other and Unspecified Psychosis (F28 & F29)]	Using mobile device for at least one year
Duration of illness ≥ 2 years	Able to read Hindi or English
Persistent significant psychopathology defined as score of ≥4 (moderate) on items P1-P7 and items N1-N7 items 7, 20, 25 or 34 on Positive and Negative Syndrome Scale (PANSS).	***Exclusion Criteria***
Persistent significant psychopathology and dysfunction, despite receiving regular treatment	Caregivers suffering from psychiatric disorder which might interfere with the care of patients and his/her co-operation during
Using mobile device for at least one year	Not willing to provide informed consent
Able to read Hindi or English	
Exclusion Criteria	
Unable to provide informed consent	

For peer review only - http://bmjopen.bmj.com/site/about/guidelines.xhtml

Supplementary Table 3 : Study Assessment Schedule for WP 4

Study Instruments	Baseline	Month 1	Month 3
**Assessment of Patients**			
Socio-demographic & Clinical Characteristics	X	X	X
Assessment of Psychopathology:	X	X	X
Positive and Negative Syndrome Scale	X	X	X
Schizophrenia Cognitive Rating Scale (SCoRS)	X	X	X
Assessment of Physical Health	X	X	X
Assessment of Treatment Adherence:	X	X	X
Medication Adherence Rating Scale (MARS)	X	X	X
Assessment of Functioning:	X	X	X
Katz Index of Independence in Activities of Daily Living (ADL)	X	X	X
Lawton Brody Instrumental Activities of Daily Living (IADL)	X	X	X
World Health Organization Disability Assessment Schedule (WHODAS)2.0	X	X	X
Clinical Global Improvement	X	X	X
Assessment of Caregivers			
Mini International Neuropsychiatric Interview (MINI) Screen for caregivers	X	X	X
Family Burden Interview Schedule	X	X	X
Ways of Coping Checklist-Hindi Adaptation (WCC-HA)	X	X	X

For peer review only - http://bmjopen.bmj.com/site/about/guidelines.xhtml

For peer review only

Section C – Work Package 5:-Early detection using a community awareness programme (Commissioned Project)

India has a young population – over half of Indian's population are under the age of 30 years (22), with an overall mental illness prevalence rate of 10.6% and nearly 150 million Indians in need of active interventions (23). Taking into account that 80% of FEP usually occurs at a very young age (i.e. between 16 and 30 years of age) (24) and that LMICs have predominantly young populations, it is highly likely that an overwhelming majority of people experiencing FEP live in LMIC countries such as India. However, lack of adequate mental health services in India along with other impediments to care such as stigma and lack of awareness can lead to delayed help-seeking contributing to long DUPs. A longer duration of DUP is associated with a poor treatment response, greater subsequent disability and higher mortality rates (25); therefore, timely identification and diagnosis are pivotal. India does not have standardized early detection and intervention programs for psychotic disorders. There are sporadic awareness and screening programs held in some schools and colleges. Moreover, these programs have not integrated active referral of "cases" to appropriate interventions. This work package therefore aims to increase mental health literacy of teachers and students; to use a web-based screening tool to detect youth who could benefit from mental health services; and to refer such youth to appropriate services.

Intervention

We will develop mental health training modules for teachers and mental health awareness modules for students. The training manual for teachers will contain information on mental illness, especially psychosis and is aimed to create awareness about the early signs and symptoms of psychosis to facilitate early referrals to clinical care. Data on Knowledge, Attitude and Practice (KAP) of the teachers and students will be obtained at baseline, month 12 and month 24. Training for teachers include one half day session (4 hours) and three subsequent booster sessions (1 hour) in three-month intervals. Students will be provided education on mental health – one half day session (4 hours). Voluntary mental health screening tool with the PHQ-9, GAD-7 and CAPE-P15 will be made available to all students through web-based applications and mobile applications. The overall research methodology is depicted in supplementary figure 3 and for the list of study instruments, see supplementary Table 4.

For peer review only - http://bmjopen.bmj.com/site/about/guidelines.xhtml

Supplementary figure 3: Research Methodology of Work Package 5

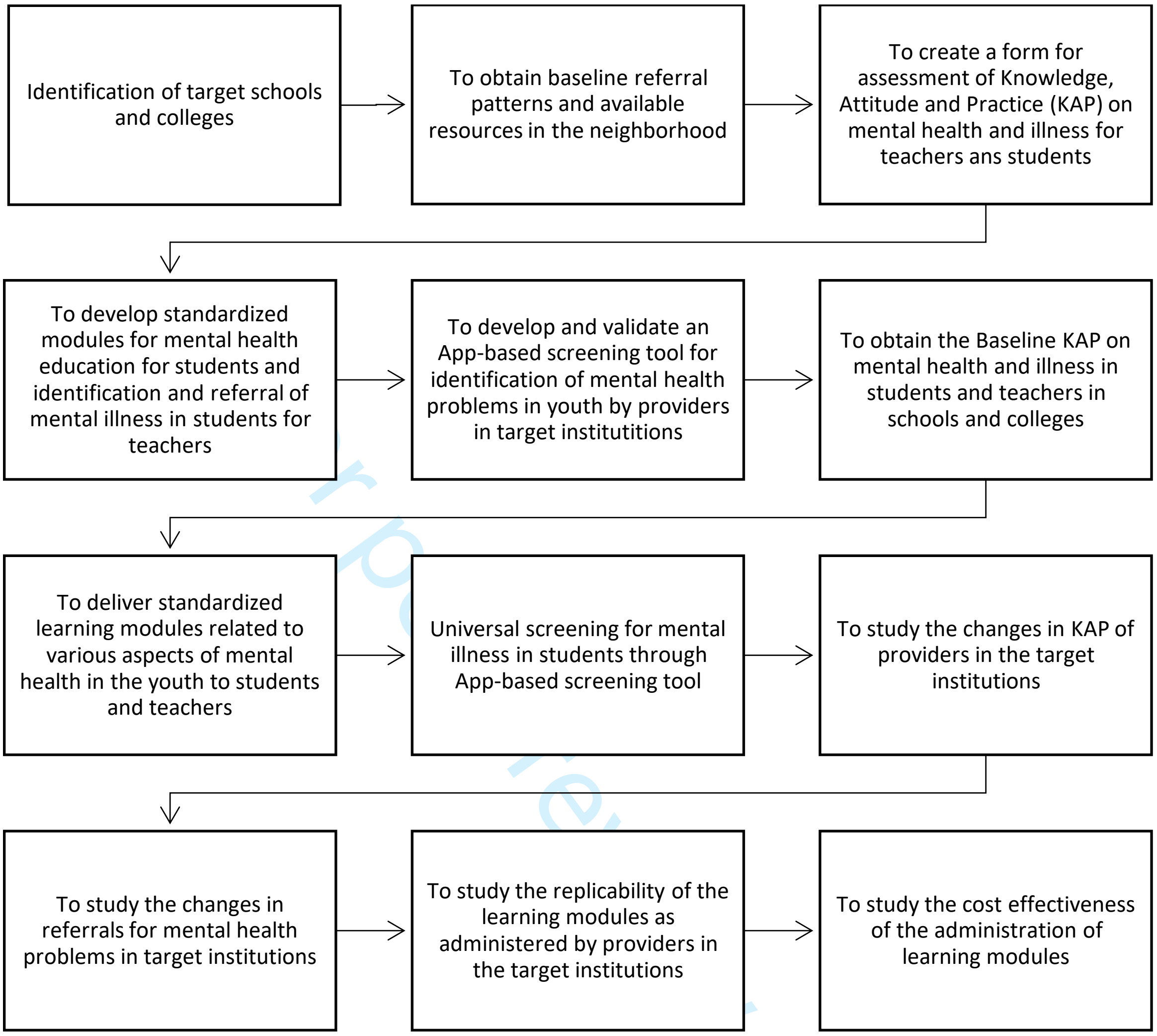

Supplementary Table 4 : List of study instruments

**Knowledge, Attitude and Practice (KAP) questionnaires**
The Mental Health Knowledge Schedule<br>The Reported and Intended Behaviour scale
**Mental health screening tool**
Patient Health Questionnaire-9 (PHQ-9)<br>Generalized Anxiety Disorder-7 scale (GAD-7)<br>Community Assessment of Psychic Experiences – Positive 15-items Scale (CAPE-P15)

For peer review only - http://bmjopen.bmj.com/site/about/guidelines.xhtml

References

1. Andreasen NC, Arndt S, Miller D, Flaum M, Nopoulos P. Correlational studies of the Scale for the Assessment of Negative Symptoms and the Scale for the Assessment of Positive Symptoms: an overview and update. Psychopathology. 1995;28(1):7-17.
2. Young RC, Biggs JT, Ziegler VE, Meyer DA. A rating scale for mania: reliability, validity and sensitivity. Br J Psychiatry. 1978;133:429-35.
3. Montgomery SA, Asberg M. A new depression scale designed to be sensitive to change. Br J Psychiatry. 1979;134:382-9.
4. Overall JE, Gorham DR. The Brief Psychiatric Rating Scale. Psychological Reports. 1962;10(3):799-812.
5. Jones SH, Thornicroft G, Coffey M, Dunn G. A brief mental health outcome scale-reliability and validity of the Global Assessment of Functioning (GAF). Br J Psychiatry. 1995;166(5):654-9.
6. Skodol AE, Link BG, Shrout PE, Horwath E. Toward construct validity for DSM-III Axis V. Psychiatry Res. 1988;24(1):13-23.
7. Herdman M, Gudex C, Lloyd A, Janssen M, Kind P, Parkin D, et al. Development and preliminary testing of the new five-level version of EQ-5D (EQ-5D-5L). Qual Life Res. 2011;20(10):1727-36.
8. Singh SP, Brown L, Winsper C, Gajwani R, Islam Z, Jasani R, et al. Ethnicity and pathways to care during first episode psychosis: the role of cultural illness attributions. BMC Psychiatry. 2015;15:287.
9. Singh SP, Cooper JE, Fisher HL, Tarrant CJ, Lloyd T, Banjo J, et al. Determining the chronology and components of psychosis onset: The Nottingham Onset Schedule (NOS). Schizophr Res. 2005;80(1):117-30.
10. Vanelli I, Chendo I, Gois C, Santos J, Levy P. [Medication adherence rating scale]. Acta Med Port. 2011;24(1):17-20.
11. David A, Buchanan A, Reed A, Almeida O. The assessment of insight in psychosis. Br J Psychiatry. 1992;161:599-602.
12. Pennebaker JW, Susman JR. Disclosure of traumas and psychosomatic processes. Soc Sci Med. 1988;26(3):327-32.
13. Mollica RF, Caspi-Yavin Y, Bollini P, Truong T, Tor S, Lavelle J. The Harvard Trauma Questionnaire. Validating a cross-cultural instrument for measuring torture, trauma, and posttraumatic stress disorder in Indochinese refugees. J Nerv Ment Dis. 1992;180(2):111-6.
14. Brugha TS, Cragg D. The List of Threatening Experiences: the reliability and validity of a brief life events questionnaire. Acta Psychiatr Scand. 1990;82(1):77-81.
15. Thara R, Padmavati R, Kumar S, Srinivasan L. Instrument to assess burden on caregivers of chronic mentally ill. Indian J Psychiatry. 1998;40(1):21-9.
16. Chadda RK. Caring for the family caregivers of persons with mental illness. Indian J Psychiatry. 2014;56(3):221-7.
17. Chadda RK, Singh TB, Ganguly KK. Caregiver burden and coping: a prospective study of relationship between burden and coping in caregivers of patients with schizophrenia and bipolar affective disorder. Soc Psychiatry Psychiatr Epidemiol. 2007;42(11):923-30.
18. Stalin P, Abraham SB, Kanimozhy K, Prasad RV, Singh Z, Purty AJ. Mobile Phone Usage and its Health Effects Among Adults in a Semi-Urban Area of Southern India. J Clin Diagn Res. 2016;10(1):LC14-6.
19. Firth J, Torous J. Smartphone Apps for Schizophrenia: A Systematic Review. JMIR Mhealth Uhealth. 2015;3(4):e102.

For peer review only - http://bmjopen.bmj.com/site/about/guidelines.xhtml

20. Sood M, Chadda RK, Singh P. Mobile health (mHealth) in mental health: Scope and applications in low-resource settings. Natl Med J India. 2016;29(6):341-3.
21. M Sood RC, K Sinha Deb, R Bhad , A Mahapatra, R Verma, AK Mishra. Scope of Mobile Phones in Mental Health Care in Low Resource Settings. Journal of Mobile Technology in Medicine. 2016(5:2:33–37).
22. Factbook CIAW. India Demographics Profile 2018 2018 [updated January 20 2018. Available from: https://www.indexmundi.com/india/demographics_profile.html.
23. Murthy RS. National Mental Health Survey of India 2015-2016. Indian J Psychiatry. 2017;59(1):21-6.
24. Shiers D, Lester H. Early intervention for first episode psychosis. BMJ. 2004;328(7454):1451-2.
25. Farooq S, Large M, Nielssen O, Waheed W. The relationship between the duration of untreated psychosis and outcome in low-and-middle income countries: a systematic review and meta analysis. Schizophr Res. 2009;109(1-3):15-23.

For peer review only
